# Bone Grafts: Everything You Need to Know

**DOI:** 10.1111/jre.70087

**Published:** 2026-02-19

**Authors:** Håvard Jostein Haugen, Javier Sanz, Giuseppe Perale, Augustine Mark Saiz, Mario Romandini, Maryam Rahmati

**Affiliations:** 1 Department of Biomaterials, Institute of Clinical Dentistry, University of Oslo, Oslo, Norway; 2 ETEP (Etiology and Therapy of Periodontal and Peri-Implant Diseases) Research Group, University Complutense (UCM), Madrid, Spain; 3 Section of Periodontology, Faculty of Odontology, University Complutense, Madrid, Spain; 4 Faculty of Biomedical Sciences, University of Southern Switzerland, Lugano, Switzerland; 5 Ludwig Boltzmann Institute for Experimental and Clinical Traumatology, Vienna, Austria; 6 Industrie Biomediche Insubri SA, Mezzovico-Vira, Switzerland; 7 Department of Orthopaedic Surgery, UC Davis Health, Sacramento, California, USA; 8 Ninth People’s Hospital, Shanghai Jiao Tong University School of Medicine, Shanghai, China

**Keywords:** allografts, autogenous bone, biomaterials, bone regeneration, bone substitutes, immunomodulation, periodontal diseases, sinus lift, synthetic grafts, xenografts

## Abstract

Bone substitute biomaterials have become a *sine qua non* in periodontology and implant dentistry; however, the ideal material choice remains controversial. In this review, we examine natural grafts (autograft, allograft, xenograft) and synthetic grafts (alloplasts, composites, CAD-CAM personalised materials), comparing them across the triad of osteogenesis, osteoinduction, and osteoconduction. After a thorough presentation of current classification, properties, and mechanisms of action of the various bone grafts, we outline their clinical applications across different indications and discuss future directions in the field. Autografts provide living cells and influential inductive factors, but at the expense of donor-site morbidity and rapid, unpredictable resorption. Processed allografts and xenografts provide reliable osteoconduction and volumetric stability with reduced inductive potential. Modern alloplasts (β-TCP, hydroxyapatite, bioactive glass) achieve outcomes comparable to those of natural grafts in selected indications, particularly for space maintenance. Composite strategies, which blend small fractions of autogenous chips with slowly resorbing xenografts or alloplasts and are protected by membranes or meshes, enhance contour stability in sinus floor elevation as well as in lateral and vertical bone augmentation (i.e., guided bone regeneration). In periodontal regeneration of intra-bony and furcation defects, non-autogenous bone grafts are added to biologics (e.g., enamel matrix derivatives, platelet concentrates) when space maintenance is needed. Bone substitute materials represent the gold standard for socket preservation, while their adjunctive benefits in peri-implantitis reconstructive surgery remain limited. CAD–CAM patient-specific scaffolds and peptide-modified matrices may enhance fit and handling, but they face challenges related to cost, manufacturing, and regulatory hurdles. The main barriers to translation include batch-to-batch variability, regulatory heterogeneity, and limited long-term safety data. Bioactive glasses are also promising, as they simulate native mineral and do not exhibit unfavourable resorption characteristics, especially when strontium is added or when they are used in combination with platelet concentrates. They, however, remain costly and have long manufacturing times, requiring strict quality control. Cost-effectiveness remains decisive: indeed, only grafts that offer procedural savings through faster healing and fewer reinterventions are likely to be widely adopted. The future of bone grafts lies in precision biomimetics that integrate intelligent drug delivery, personalised design, and rigorous quality control, promising to extend clinical outcomes beyond the traditional autograft paradigm.

## 1 | Introduction

The field of bone-replacement materials is complex and multifaceted, currently a key component of modern periodontology and implant dentistry [1]. Whether the challenge is to reconstruct a deficient ridge, regenerate a periodontal defect, or provide an appropriate socket for a future implant, clinicians are increasingly considering using substitute graft materials to replace what has been lost due to disease, trauma, or congenital malformation. This is not a simple task: patients are living longer, periodontal diseases and caries remain highly prevalent, and accidents are still taking teeth and bone out of the equation [2–4]. Successful bone regeneration depends on three fundamental biological processes [5]. Osteogenesis involves the formation of new bone by living cells, primarily osteoblasts, which synthesise the bone matrix and initiate mineralisation. Osteoinduction represents the recruitment and differentiation of pluripotent stem cells into bone-forming cells through biochemical signalling pathways, often mediated by growth factors such as bone morphogenetic proteins (BMPs). Osteoconduction provides the structural framework that guides bone growth and vascularisation, requiring materials with appropriate porosity, surface chemistry, and mechanical properties [6]. In all these cases, the well-selected graft serves as a temporary scaffold to direct cell migration, vascular ingrowth, and, ultimately, the deposition of new bone [7]. After reviewing current challenges in bone graft biomaterials and providing a thorough presentation of the classification, properties, and mechanisms of action of various grafts, this review outlines their clinical applications across different indications and discusses future directions in the field.

## 2 | Classification of Bone Grafts

**FIGURE 1 |:**
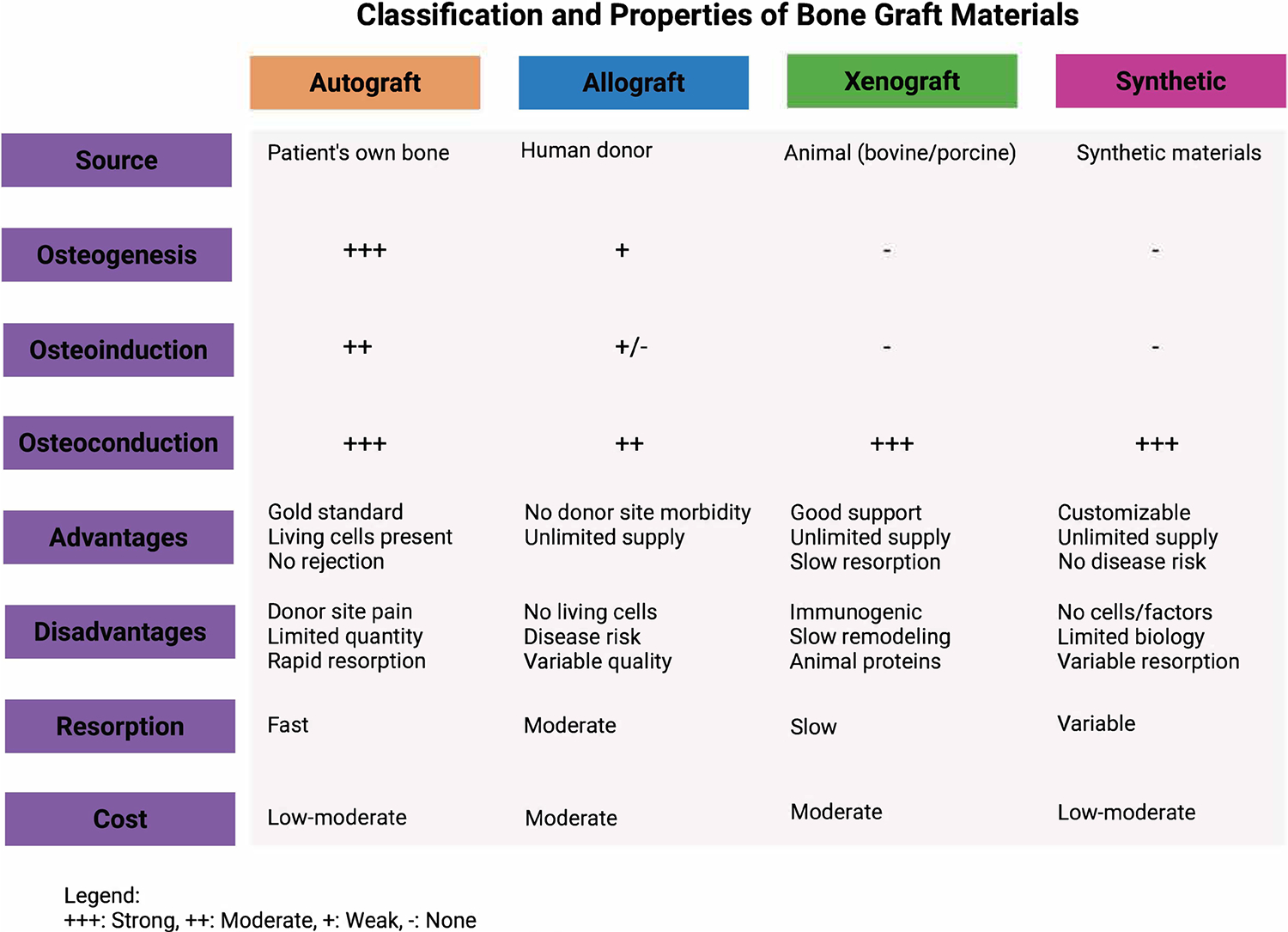
Classification and properties of bone graft materials. Comprehensive comparison of the four main categories of bone graft materials used in periodontal and implant dentistry. The table illustrates key distinguishing features, including biological source, regenerative properties (osteogenesis, osteoinduction, and osteoconduction), clinical advantages and disadvantages, resorption kinetics, and relative cost. Colour-coded cells indicate the strength of each biological mechanism: Green (+++, strong), gold (++, moderate), orange (+/−, weak), and pink (−, absent). Autografts represent the “gold standard” for all three biological properties but are limited by donor-site morbidity and the limited supply of donors. Allografts and xenografts provide osteoconductive scaffolds that do not contain living cells. Alloplasts (synthetic materials) offer unlimited availability and customisability but lack inherent biological activity. This classification framework guides clinical decision-making based on defect characteristics, patient factors, and treatment objectives.

Bone grafts are often categorised into two groups: natural and synthetic, each offering distinct benefits and drawbacks. Natural grafts, encompassing autografts, allografts, and xenografts, are sourced directly from biological tissue. Figure 1 summarises the classification of bone grafts and their key properties.

### 2.1 | Autografts

Autografts are transplants from one location to another within the same patient and currently represent the ideal bone graft material because they combine all three crucial characteristics for repair: viable osteogenic cells (osteogenesis), inductive growth factors (osteoinduction), and a calcified matrix scaffold (osteoconduction) [8]. Donor sites commonly include extraoral locations, such as the iliac crest, tibia, and calvaria, or intraoral sites such as the mandibular ramus, symphysis, or local bone, depending on the clinical indication and the required graft volume [9–11]. However, autograft retrieval results in the necessity of a supplementary surgical procedure that augments operating time and morbidity, with possible complications such as pain, infection, or bleeding [12].

The harvesting method is critical and directly influences graft viability and biological performance. Moreover, cells survive better during harvesting when no mechanical trauma or desiccation is involved [13]. Processed graft resorption is hastened, with loss of native growth factors (including BMPs, TGF-β, and VEGF) in conventionally harvested GBR sites compared to less traumatic ones [10]. These limitations, along with donor-site morbidity and variability in resorption rates, limit the applicability of autografts to significant defects [14].

### 2.2 | Allografts

Allografts refer to bone grafts obtained from a donor of the same species as the recipient, but who is genetically different [15, 16]. Allograft bone is typically procured from human cadavers via tissue banks, though it can also come from living donors (for instance, surplus bone from orthopaedic surgeries). Allograft is the most commonly used type of bone graft worldwide [8]. This popularity is mainly due to the absence of a second surgical site and the ability to provide substantial volumes of graft material on demand.

Allograft bone is available in various preparations and formats to suit clinical requirements. For example, it can be supplied fresh or, more commonly, as processed tissue—such as fresh-frozen bone, freeze-dried bone allograft (FDBA), or demineralised bone matrix (DBM) – and in various forms, ranging from structural cortical blocks and whole bone segments to cancellous chips and paste-like putties [17]. Autografts contain viable osteoblasts and osteoprogenitor cells that survive transplantation through rapid revascularisation from the recipient bed, enabling early bone matrix deposition and integration via creeping substitution [18]. The mineralised matrix serves as a natural reservoir of growth factors, including bone morphogenetic proteins (BMPs), transforming growth factor-β (TGF-β), and vascular endothelial growth factor (VEGF), which are gradually released during resorption and remodelling [19]. Together, these cellular and molecular mechanisms create a highly bioactive environment that supports coordinated osteogenesis, angiogenesis, and defect stabilisation.

Each processing method affects the biological and handling properties of the graft. Fresh or frozen allografts retain greater levels of native proteins and growth factors; thereby, they may offer greater osteoinductive potential. However, they carry a higher risk of immune rejection and disease transmission and are less convenient to store [20, 21]. Freeze-dried bone has a significantly longer shelf life and reduced immunogenicity, albeit at the expense of diminished osteogenic, osteoconductive, and mechanical strength [22].

Despite their widespread use, allografts have significant limitations compared to autografts [23]. Allograft tissue is foreign to the recipient’s immune system. Even with careful donor screening and laboratory testing, a small risk of transmitting infections, such as viruses or prion diseases, from donor to host remains a concern [24]. Moreover, the recipient’s immune system may mount an immune response to residual proteins in the graft. Banked allograft bone is typically devitalised and sterilised to minimise these risks through deep-freezing, gamma irradiation, chemical deproteination, and/or demineralisation [25]. While these processes make allografts relatively safe and biologically inert, they also remove living cells and reduce or eliminate certain osteoinductive factors. As a result, allografts primarily serve as an osteoconductive scaffold; they lack the inherent osteogenic capacity of autografts and exhibit substantially less osteoinductive activity [26, 27]. Therefore, autografts are primarily osteogenic and osteoconductive, with an indirect, inconsistently demonstrated osteoinductive potential. Clinically, the long-term incorporation of allograft bone is less predictable. Studies have reported that allografts have higher long-term failure and non-union rates than autografts [28, 29]. These failures often relate to the allograft’s delayed or incomplete remodelling and its susceptibility to resorption or fracture over time.

Another concern with allografts is their limited availability and the regulatory burden associated with them. In many regions, demand for allograft tissue exceeds the supply of donor bone, leading to shortages in tissue banks. Stricter regulations, such as the European Tissue and Cells Directive [30] and the 2017 medical device regulations have imposed more rigorous requirements on the procurement, processing, and clinical use of human tissue [31]. Compliance with these regulations increases the cost and complexity of using allografts, and in some cases, clinicians have moved away from allograft use in favour of other graft substitutes [32]. Despite these challenges, allografts remain a mainstay in bone grafting practice due to their convenience and elimination of donor-site morbidity.

**FIGURE 2 |:**
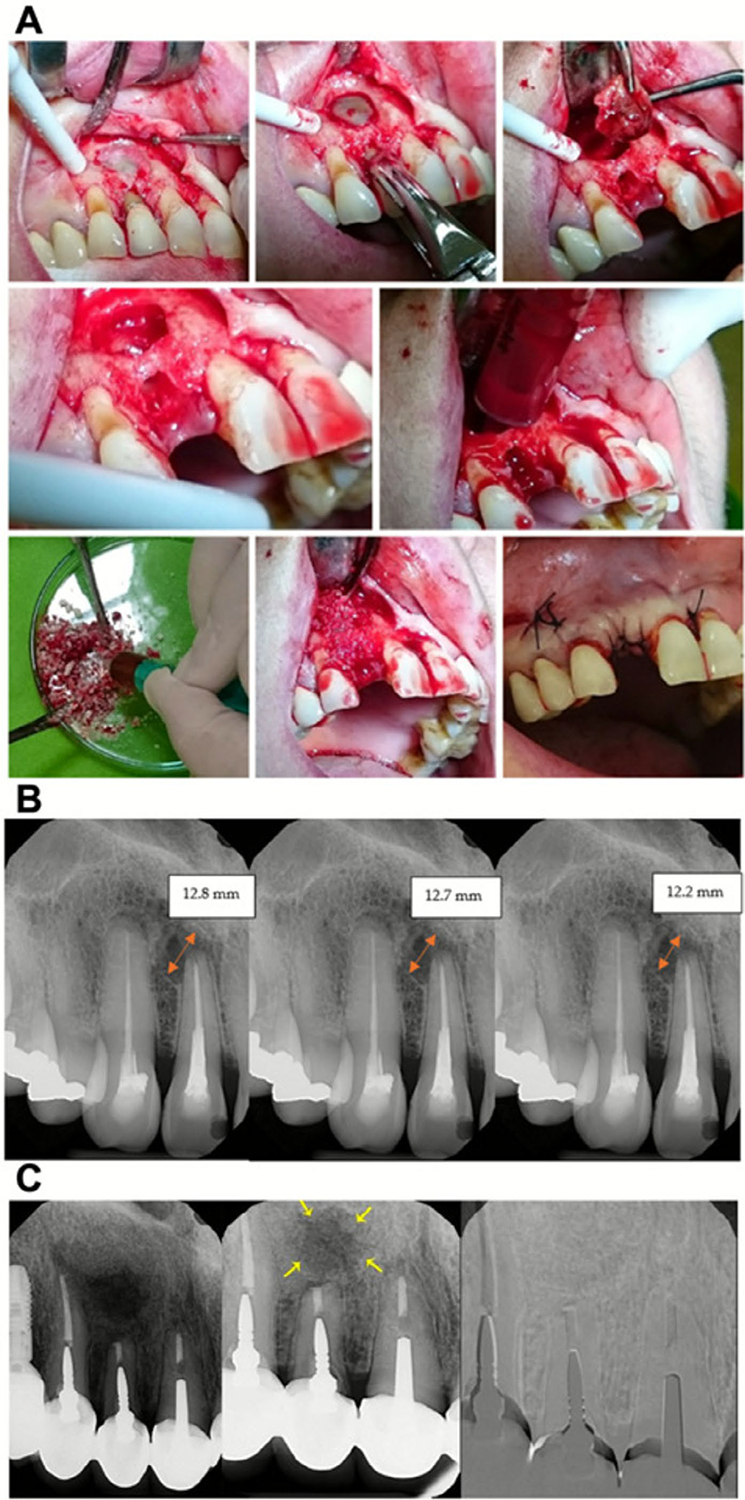
(A) Intraoperative photographs showing odontogenic cyst enucleation, defect preparation, and placement of albumin-impregnated bone allograft (BoneAlbumin) in the maxillary anterior region. Sequential periapical radiographs of ungrafted controls (B) and albumin-impregnated allograft group (C) taken immediately post-op, at 6 weeks, and 12 weeks, demonstrating significantly accelerated defect healing in the albumin group. Figure published by [33] MDPI, Basel, Switzerland under the terms of the Creative Commons Attribution 4.0 International Licence (CC BY 4.0), which permits unrestricted reproduction.

Ongoing improvements in processing aim to enhance allograft safety and performance, but the search for alternatives highlights the need to address their biological shortcomings. Recent innovations have explored the functionalisation of allografts to improve their regenerative capacity. Rangics et al. [33] conducted a randomised clinical trial in patients with maxillary odontogenic cysts, comparing albumin-impregnated bone allografts (BoneAlbumin) with ungrafted controls. The study found significantly greater defect size reduction in the albumin group by 6 weeks (*p* < 0.000001) and 12 weeks (*p* = 0.000296), indicating that albumin coating accelerates early bone healing. Surgical images (Figure 2A) demonstrate the cyst enucleation and graft placement workflow, while sequential radiographs (Figure 2B) and quantitative measurements (Figure 2C) clearly show faster and more complete defect closure in the albumin group. Together, these results highlight albumin-coated allografts as a promising strategy to enhance allograft osteoinductivity without relying on living cells [33].

### 2.3 | Xenografts

Xenografts are bone grafts derived from a donor of a different species than the human recipient. Xenografts are most sourced from bovine bone (e.g., DBBM), but porcine-derived alternatives have garnered attention as their use becomes more widespread. A recent systematic review and meta-analysis of 202 grafted sites found no significant differences in new bone formation, radiographic gain, or histomorphometric outcomes between porcine and bovine xenografts in sinus floor augmentation and ridge preservation. These findings support the clinical interchangeability of porcine- and bovine-based xenografts, providing clinicians with more flexible options for xenografts [34]. A split-mouth RCT by Cha et al. [35] involving bilateral sinus floor augmentations found no significant differences in histologic new bone formation, residual graft volume, or bone-to-graft contact between bovine (Bio-Oss) and porcine (GenOs) xenografts at 6 months post-op. Both materials exhibited similar clinical handling characteristics and achieved 100% implant survival [35].

Xenografting provides access to a virtually unlimited supply of graft material, as animal bones can be harvested in large quantities. The mineral composition of bovine bone closely resembles that of human bone, making it an effective natural scaffold for regenerating human bone. Bovine xenograft materials (often in the form of deproteinised bone mineral) have become especially popular in dentistry and cranio-maxillofacial surgery for ridge augmentation and sinus lift procedures [36]. These grafts serve as an osteoconductive framework, allowing the patient’s bone to gradually grow into them. Over time, xenograft particles may become surrounded by or fused with new bone, though complete replacement by host bone is typically slow and may remain incomplete even years after implantation [14].

The use of xenografts, however, presents several biological challenges. Because the donor species differs, there is an inherent risk of immune rejection, as the human immune system can recognise animal proteins as foreign [22]. There is also a hypothetical risk of cross-species disease transmission—for example, viruses or prions that affect the donor species could infect humans. Decades of clinical experience and studies have, however, reported no occurrences of prion disease transmission from commercial bovine bone grafts, and such materials are generally considered safe for routine use [24]. Modern xenograft preparations mitigate these risks by extensive processing, often including high-temperature sintering and a purification step [37].

Bovine bone xenografts are typically thoroughly deproteinised, removing all cells and organic components, leaving only the mineral matrix, either by heat or chemical treatment [5]. These steps eliminate living cells, microbes, and immunogenic proteins from the graft, resulting in a final product that is essentially an inorganic calcium phosphate lattice [5]. This process yields a biocompatible, biologically inert material, meaning it is osteoconductive but not osteoinductive. The lack of viable cells and growth factors in xenografts means they rely entirely on the host environment for new bone formation [38]. Additionally, residual xenogeneic collagen or other proteins (if any remain) could elicit immune responses; however, this is minimised in well-processed bovine bone grafts.

Isolated reports have described low-grade inflammatory or foreign-body responses to xenogeneic granules [39]. Yet, serious sequelae are uncommon, and bovine xenografts remain a dependable mainstay, particularly where access to human donor bone is limited, despite sometimes facing ethical objections to the use of animal-derived products and religious restrictions.

### 2.4 | Alloplasts (Synthetic Grafts)

Alloplasts are synthetic bone graft substitutes, meaning they are man-made materials (inorganic or organic) designed to fulfil the role of bone grafts. Unlike autografts, allografts, or xenografts, which originate from biological tissue, alloplastic grafts are created from non-living materials such as ceramics, polymers, metals, or composites. The most used alloplasts in bone regeneration are calcium phosphate-based ceramics, including hydroxyapatite (HA) and β-TCP. These materials closely mimic the mineral component of bone. They are frequently supplied as granules, blocks, or porous scaffolds that can be placed into bone defects. Other examples of alloplasts include bioactive glass (a silica-based material that can bond to bone), calcium sulphate (plaster of Paris), and various biodegradable polymers (e.g., polylactic acid or polycaprolactone scaffolds). Even metallic implants with porous structures or 3D-printed scaffolds made of novel biomaterials fall under the category of alloplastic bone substitutes [40]. Alloplasts are readily available in virtually unlimited quantities and in pre-shaped forms, eliminating the need for donor-site surgery and the supply constraints associated with biological grafts [19]. They also carry no risk of transmitting infections between individuals and typically evoke minimal immune response, as they are designed to be biocompatible [41].

The primary mechanism by which synthetic grafts assist bone healing is osteoconduction [42]. An alloplastic graft serves as a physical scaffold, maintaining the defect site and allowing the patient’s bone cells to migrate in and gradually replace the material [43]. Many alloplasts are porous or trabecular, facilitating vascular ingrowth and bone deposition. Most alloplasts are biologically inactive because they neither actively recruit bone-forming cells nor independently induce new bone formation [44]. They generally lack inherent osteoinductive factors and contain no living cells that could direct osteogenesis. This means that bone regeneration with alloplasts can be slower or less complete than with an autograft, unless biological stimulation is added [45]. The body’s response to different synthetic materials can vary. Some ceramics, such as β-TCP, are designed to resorb over several months and be gradually replaced by new bone [46, 47].

In contrast, others, like dense hydroxyapatite, are very slow to resorb and may remain a permanent scaffold integrated with the bone [48–50]. The degradation products of alloplasts must also be biocompatible; for instance, calcium phosphate ceramics resorb to release calcium and phosphate ions, which can be reused in the process of bone remodelling [48]. Biodegradable polymers break down into acids (such as lactic acid), which, if generated too quickly, can temporarily lower the local pH and potentially inhibit bone formation [51]. Careful material design, such as using copolymers or incorporating essential compounds, can mitigate such issues.

The clinical performance of alloplastic grafts has improved significantly over the past few decades to the point where they can achieve outcomes comparable to those of natural grafts in some instances. They are handy for filling smaller contained defects or augmenting bone around implants and are often combined with growth factors or bone marrow aspirate to enhance their activity. Alloplasts are considered a major class of graft material in modern classifications [52]. However, because they usually provide only a scaffold, their success depends heavily on the host’s regenerative capacity. In healthy patients with adequate blood supply and without large segmental bone loss, alloplasts can be very effective. In more challenging scenarios (large defects, poor biology), clinicians often use alloplasts in combination with other strategies, resulting in composite grafts.

### 2.5 | Composite Grafts and Growth Factor-Enhanced Composites

Composite grafts are combinations of two or more different types of bone graft materials or adjuncts engineered to leverage the advantages of each component while mitigating their drawbacks [53]. The rationale behind composite grafts is that a single material rarely possesses all the ideal properties for bone regeneration; therefore, a mixture can create a more effective overall graft. In practice, composites can take many forms. A typical example is combining an osteoconductive scaffold (such as an alloplastic ceramic or deproteinised bovine bone xenograft) with osteogenic cells or osteoinductive factors [54]. This could be as simple as mixing autogenous bone (which provides living cells and growth factors) with a larger volume of allograft or synthetic bone substitute (which includes bulk and structure) [19, 55]. Even a small amount of autograft added to an alloplast can significantly enhance healing, as the autograft provides osteogenic and inductive factors that the synthetic material lacks—a recent case report by Kloss et al. [56] demonstrated the clinical utility of composite grafts combining a hyaluronate-enhanced bovine xenograft (Cerabone Plus) with human allogeneic bone granules (Maxgraft) for alveolar ridge augmentation. Clinical and radiographic follow-up confirmed successful bone regeneration, stable soft- and hard-tissue dimensions, and favourable conditions for implant placement, highlighting how composite strategies can merge the volume stability of xenografts with the remodelling potential of allografts to achieve effective ridge reconstruction [56].

Ceramic-polymer composites represent the most extensively studied category of composite bone grafts, combining the mechanical strength and osteoconductive properties of ceramics with the biodegradability and processing versatility of polymers [47]. Beta-tricalcium phosphate (β-TCP) blended with poly(lactic-co-glycolic acid) (PLGA) exhibits exceptional performance in critical-size defects, with the ceramic component providing immediate structural support. At the same time, the polymer matrix controls degradation kinetics [57]. These systems achieve bone formation rates of 25%–28% in clinical applications, representing significant improvements over single-material approaches [58, 59].

The most advanced strategies in the design of composite grafts, therefore, involve solutions that incorporate bioactive molecules to improve the scaffolds’ biological performance via directed molecular signalling. Of these, recombinant human bone morphogenetic protein-2 (rhBMP-2) has been the most widely investigated and is among the few growth factors approved for clinical use in bone repair [60, 61]. Bone formation achieved with rhBMP-2-loaded calcium phosphate carriers is equivalent to autografts, without donor-site morbidity [62, 63]. But recombinant BMPs can be used at supra-physiological concentrations in orthopaedic and maxillofacial areas [64, 65]. They could lead to dose-dependent side effects such as ectopic bone formation, inflammation, and oedema. For this reason, the clinical use of rhBMP-2 systems should be approached with great caution, with attention to growth factor loading, release kinetics, and carrier properties to achieve predictable therapeutic efficacy without unwanted side effects [28, 66].

Concomitantly, recombinant human platelet-derived growth factor-BB (rhPDGF-BB) has been developed as a biologic alternative with an orthogonal mechanism of action. In contrast to rhBMP-2, a direct inductor of osteogenic differentiation, rhPDGF-BB mainly stimulates angiogenesis and fibroblast proliferation, which augment the recruitment of osteoprogenitor cells—mechanisms favouring the early phases of bone repair and wound healing [67, 68]. In periodontal and peri-implant regeneration, rhPDGF-BB, in combination with β-tricalcium phosphate or collagen carriers, has been successfully applied in clinical procedures, demonstrating better soft-tissue integration and reduced inflammation compared with rhBMP-2 [69, 70]. However, even when rhPDGF-BB is used to modulate the healing environment and enhance initial bone formation via mechanisms (II) (cell recruitment/vascularisation), its effects typically are less intense than those seen with rhBMP-2 as a consequence of being more appropriately classified as a recruiting agent/vasculogenic factor instead of an osteo-inductive stimulus [67, 68].

**FIGURE 3 |:**
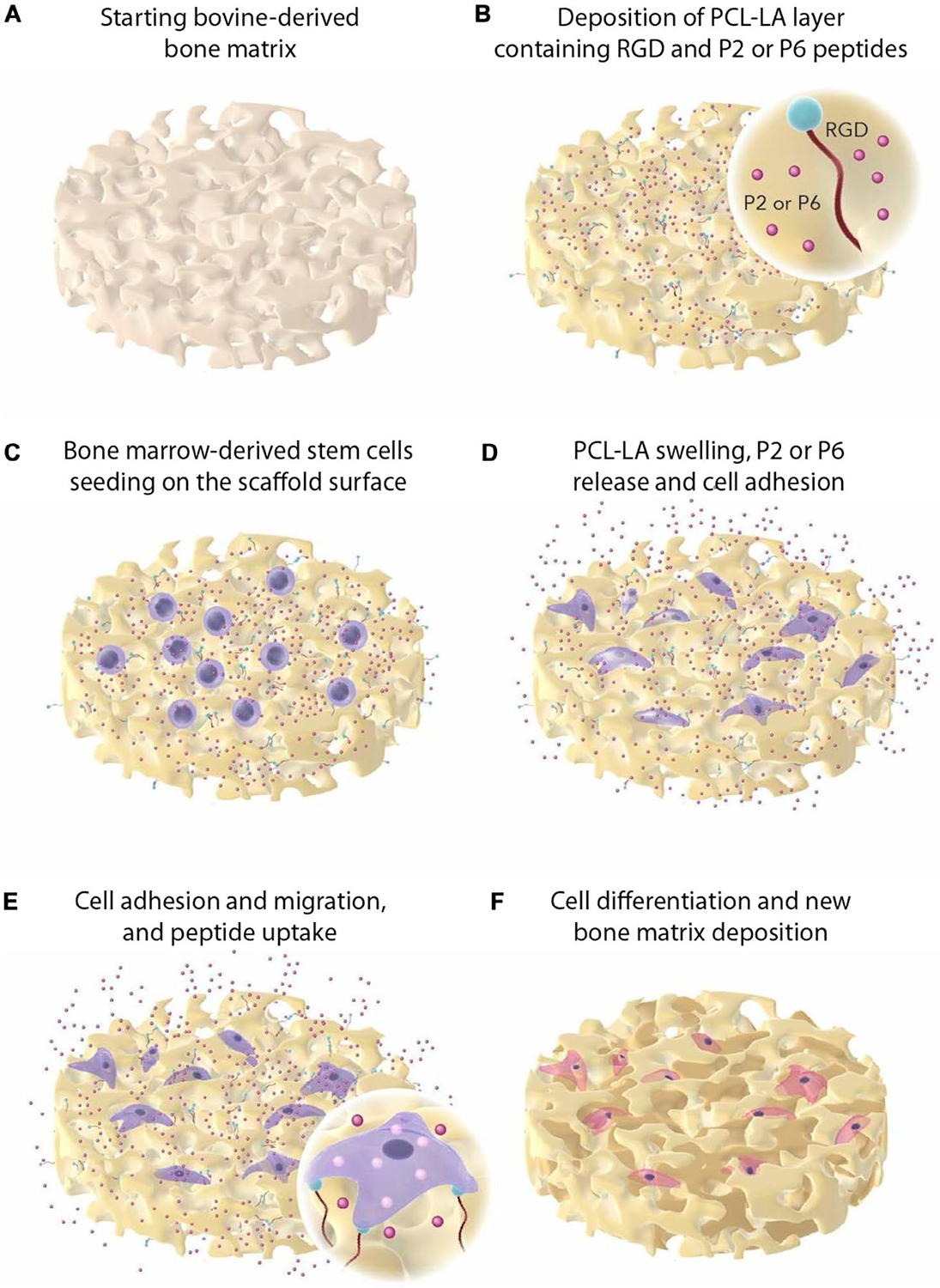
Schematic of the study concept: Peptides P2 and P6 (red spheres) are either solubilised in or physically entrapped by a poly(ε-caprolactone-lactide) (PCL-LA) coating applied to a bone graft. At the same time, mesenchymal stem cells are seeded onto the graft surface. Hydration-induced swelling of the PCL-LA layer triggers the gradual release of peptides. Adapted from Zhu et al. [75], reproduced under the Creative Commons Attribution 4.0 International Licence (CC BY 4.0), which permits unrestricted reproduction in any medium.

Alternatives to rhBMPs and rhPDGF-BB. They are synthetic molecules. One promising candidate is proline-rich intrinsically disordered proteins (IDPs), which have shown potential to influence cellular and molecular interactions critical to bone formation. Recently, two particularly promising synthetic IDPs, named P2 and P6 [71, 72] were integrated into a xenohybrid bone graft, SmartBone (SBN), to evaluate their effects on bone regeneration and growth in vivo. Previous in vitro studies indicated that these IDP-enhanced composites have a positive impact on human osteoblasts and mesenchymal stem cells derived from human bone marrow [73, 74]. In the calvarial pig model, IDPs demonstrate significant potential in enhancing both early and late stages of bone healing and biomineralisation (Figure 3) [76]. More recently, the osteogenic P2 has also been incorporated into injectable hyaluronic-acid hydrogels for polytrauma fractures, where HA + P2 (±MSCs) dampened systemic cytokine elevations towards baseline and improved early mineralisation relative to HA alone. This supports IDPs as modular, synthetic “one-to-many” cues that can be integrated across carrier chemistries to provide sustained osteogenic signalling without relying on supraphysiological recombinant growth-factor dosing douglas [77, 78].

Insulin-like growth factor-1 (IGF-1) enhanced xenografts demonstrate improved osteoblast proliferation and enhanced expression of bone formation markers, including osteocalcin and osteopontin [79, 80]. Gene-activated bone substitutes represent the newest frontier in composite technology, utilising naked plasmid DNA encoding vascular endothelial growth factor to promote both osteogenesis and angiogenesis simultaneously [63].

In clinical use, several composite graft products and techniques have shown promise. Demineralised bone matrix (DBM) putties often incorporate a carrier substance (such as collagen or glycerol), a composite of allograft-derived growth factors with a synthetic or organic carrier. Likewise, combining xenograft granules with autologous platelet-rich plasma (PRP) or other blood-derived products enhances the biological activity of the graft site [81]. Certain composites can achieve bone healing outcomes comparable to those of autograft alone [82, 83]. Autologous platelet concentrates, particularly L-PRF, significantly enhance bone and soft tissue healing in periodontal regenerative procedures, demonstrating clear benefits in alveolar ridge preservation, sinus floor augmentation, and the regeneration of periodontal defects [84, 85]. Among APCs, L-PRF consistently provides superior clinical outcomes, is easy to prepare, and is completely autologous, making it an ideal choice for personalised regenerative treatments [85].

**FIGURE 4 |:**
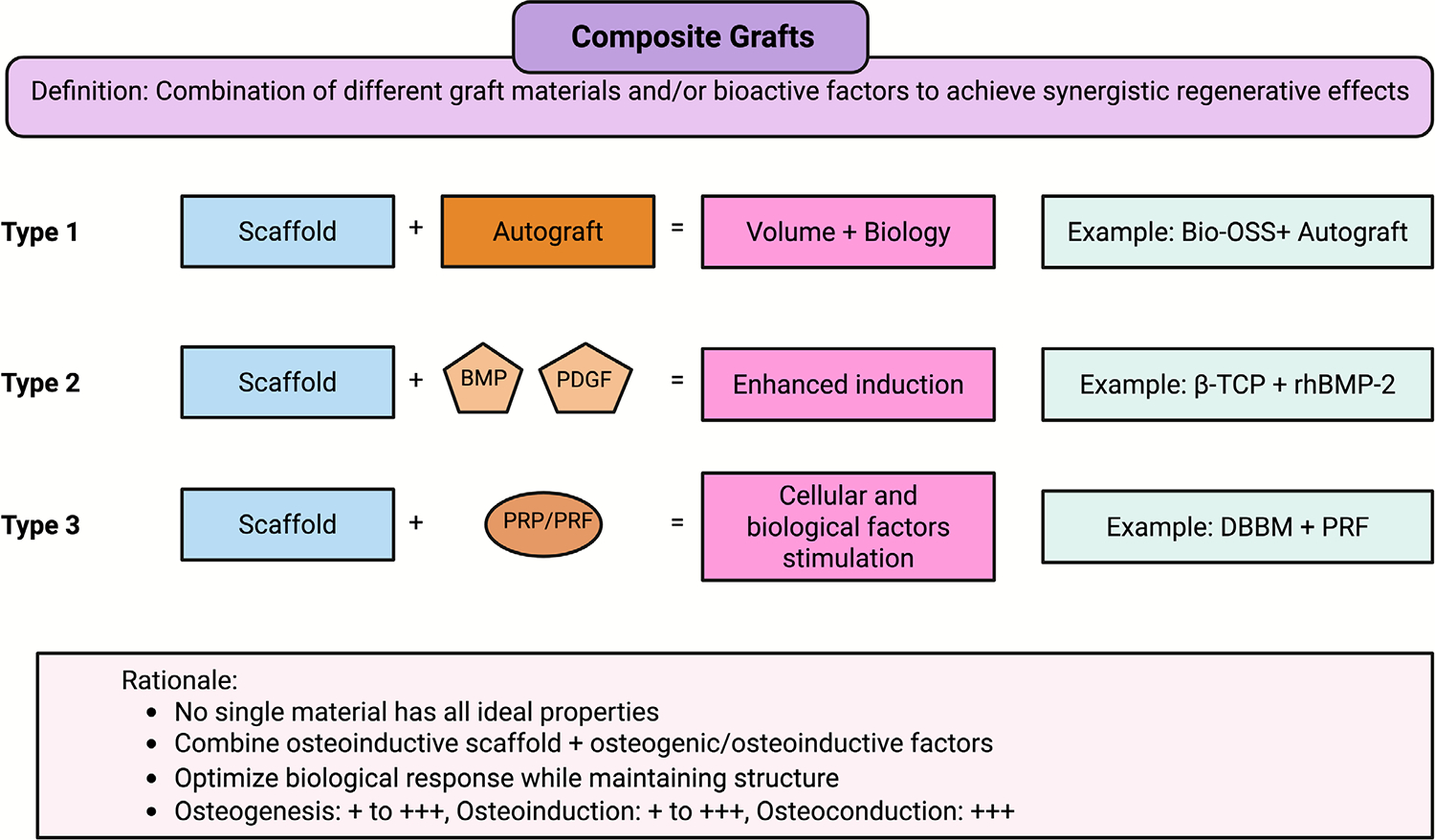
Composite bone grafts: Synergistic combinations for enhanced regeneration. Composite grafts combine two or more materials or bioactive factors to optimise biological response. Three main strategies are illustrated: (1) scaffold + autograft (combines osteoconductive volume with osteogenic cells and growth factors), (2) scaffold + recombinant growth factors such as BMP-2 or PDGF (enhances osteoinduction without donor site morbidity), and (3) scaffold + platelet concentrates (PRP/PRF deliver autologous growth factors and cells). Biological properties vary (+ to +++) depending on the specific combination, allowing clinicians to tailor grafts to the characteristics of the defect and patient factors. Clinical applications include periodontal intrabony defects, sinus floor augmentation, and alveolar ridge preservation. Examples include Bio-Oss + autograft, β-TCP + rhBMP-2, and DBBM + PRF.

The versatility of composite grafts makes them a powerful approach in modern bone regeneration. Ongoing research continues to explore new combinations, such as the incorporation of anti-inflammatory agents or angiogenic factors, to improve outcomes (Figure 4) further. This emphasis on ongoing research underscores the field’s dynamic nature and the potential for future advancements in bone regeneration.

### 2.6 | CAD-CAM Personalised Grafts

**FIGURE 5 |:**
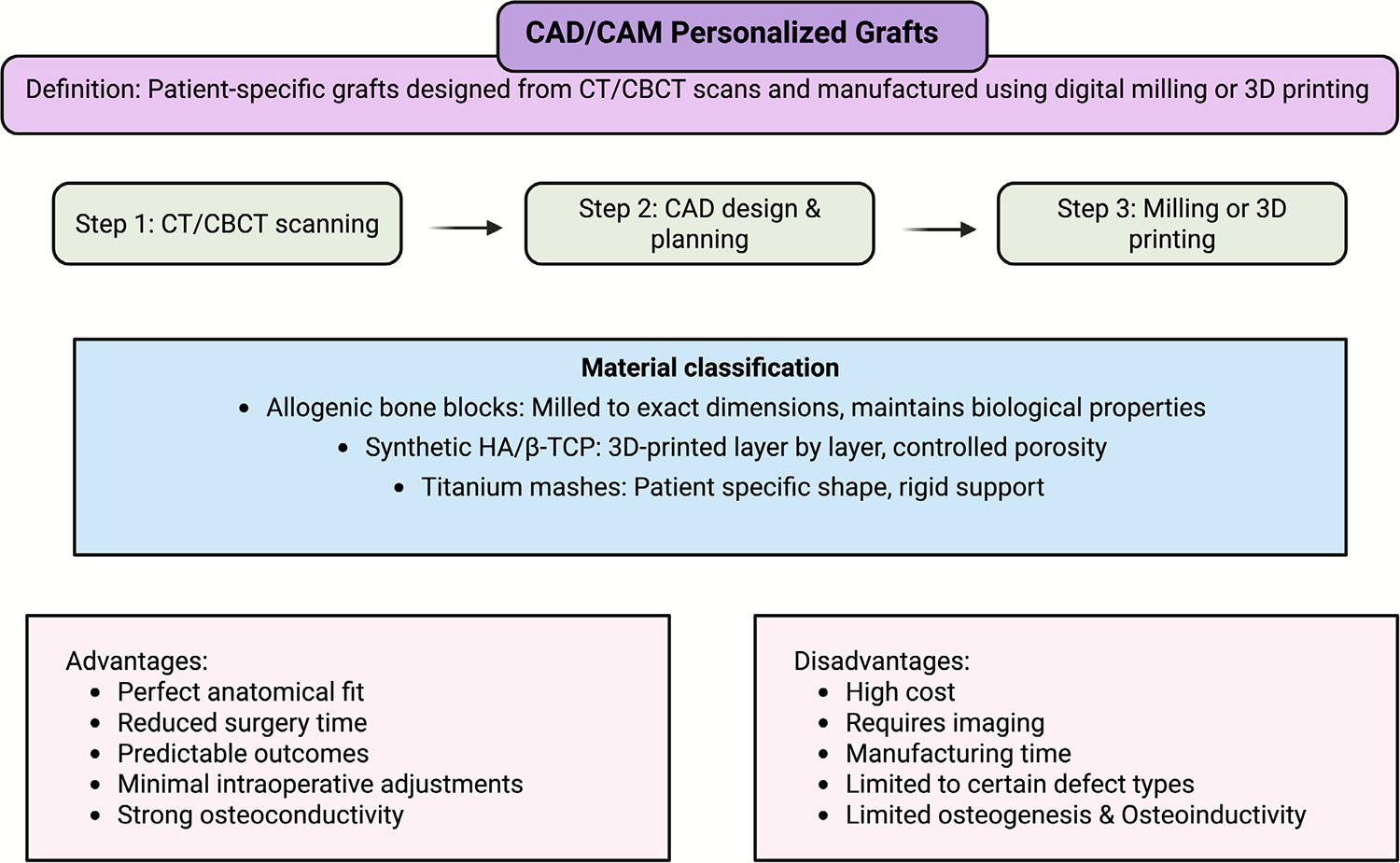
CAD-CAM personalised bone grafts: Digital workflow for patient-specific reconstruction. Patient-specific grafts are designed from CT/CBCT scans using computer-aided design and manufacturing. The three-step workflow includes: (1) three-dimensional imaging acquisition, (2) virtual surgical planning and graft design, and (3) manufacturing via milling (allogenic bone blocks) or 3D printing (synthetic HA/β-TCP or titanium meshes). Materials differ in their biological properties and structural characteristics. Advantages include perfect anatomical fit, reduced surgical time, minimal intraoperative adjustments, and predictable outcomes. Disadvantages include high cost, complex imaging and manufacturing requirements, and long lead times. These grafts are primarily osteoconductive and often combined with osteogenic/osteoinductive materials for optimal regeneration. Applications include complex three-dimensional defects, large vertical onlay grafts, and customised ridge augmentation. Examples include Yxoss CBR, SmartBone, and custom titanium meshes.

Recent technological advances in medical imaging and production have led to the advent of computer-assisted design and manufacturing (CAD-CAM) in bone grafting. CAD-CAM personalised grafts are graft substitutes that are precisely fitted to the patient’s defect and produced to size and shape (Figure 5) [86]. While an off-the-shelf graft arrives in standard shapes and sizes, a personalised graft is designed *in silico* based on the patient’s anatomy (usually obtained from a CT or MRI scan) and a three-dimensional model of the defect [87]. This digital model is used to produce a graft of the corresponding size and shape. This fabrication can be performed either by milling or carving a block (such as a cadaveric allograft bone or a synthetic ceramic block) to match the exact geometry (e.g., milling a block) or by a 3D printer that produces the scaffold skeleton layer by layer according to the geometry [88].

Allogenic bone or artificial materials are commonly used for CAD-CAM grafts because the machine can efficiently process these [89, 90]. For example, patient-specific allograft bone blocks are manufactured by milling to shape the transplanted bone and fit complex bone defects in the jaws [89]. In addition, custom porous titanium or hydroxyapatite implants can be 3D printed for the restoration of large craniofacial defects [91]. Among them, dental implantology is one of the most remarkable applications, where custom-made β-TCP/HA synthetic blocks are used to reconstruct atrophied ridges. This enables surgeons to benefit from a satisfactory graft adaptation, which facilitates the surgical process and provides a better grip on the graft during healing [92]. Supporting this approach, a recent retrospective clinical study demonstrated that custom-made CAD-CAM synthetic HA/β-TCP onlay grafts effectively regenerated small alveolar ridge defects before implant placement. Over eight months, these grafts achieved significant vertical and horizontal bone gains, exhibited excellent adaptation to the patient’s anatomy, minimised intraoperative adjustments, and resulted in high implant survival with low complication rates. These findings highlight the promise of CAD-CAM-fabricated synthetic grafts as a predictable and efficient solution for localised ridge augmentation, thereby reducing surgical time and improving precision [93].

The personalised nature of these grafts may have several advantages. The ideal fit will provide close apposition between the graft and host bone, potentially promoting osteointegration and limiting the risk of graft movement or non-union [94, 95]. It also eliminates or reduces the need for the surgeon to trim or shape the graft during surgery, in the less-than-optimal operating-table setting, thereby saving operative time and enabling more precise reconstruction of the bone to its original anatomy [96]. Furthermore, the internal structure can be optimised by designing the graft. For instance, with CAD programming, one can introduce channels or lattices into the mesh of a 3D-printed scaffold to promote vascular ingrowth or optimise material density across different regions to meet mechanical demands [97]. As CAD-CAM reconstructed bone grafts are still in the early stages of application, short-term clinical outcomes have demonstrated their effectiveness [98]. Individualised scaffolds have been shown to integrate and restore anatomical continuity, with excellent form and fit, in patients. For complex mandibular or cranial reconstructions, these methods enable functional and aesthetic results that are difficult to achieve with standard graft shapes [99]. However, this method also has obstacles: design and production can take years and cost millions of dollars, sometimes involving expensive equipment and a team of surgeons, radiologists, and bioengineers [100]. Furthermore, the bioactivity of a specifically designed implant still relies on its material; an individually designed artificial block remains an artificial one, with the associated lack of bioactivity [101, 102]. As a result, some individually tailored grafts are employed with biological modifications (i.e., coating the personalised graft with BMPs or seeding with the patient’s cells prior to implantation) [103–105]. With the ongoing development of 3D printing and computer-based surgical tools, personalised bone grafts may increasingly be used, further expanding the clinician’s toolkit for repairing skeletal defects in accordance with each patient’s specific needs.

### 2.7 | Resorbable Versus Non-Resorbable Bone Grafts

Building on the discussion of CAD-CAM personalised grafts in the previous section, it is essential to emphasise that the clinical behaviour of such patient-specific constructs is determined not only by their geometric precision but also by the intrinsic resorption profile of the underlying material. Whether the personalised graft is milled from an allogenic block or 3D-printed from a synthetic ceramic or titanium scaffold, its biological performance depends fundamentally on whether the chosen material is resorbable or non-resorbable. Thus, material degradability represents a key parameter in the design and clinical indication of CAD-CAM grafts, influencing long-term stability, integration, and regenerative potential.

One further criterion for classifying bone grafts is therefore their degree of resorption and retention of structural integrity. Biodegradable grafts—including autografts, allografts, and many synthetics such as β-tricalcium phosphate (β-TCP)—undergo gradual degradation in vivo and are replaced by newly formed bone over several months. Their resorption dynamics, however, can be variable and influenced by composition, porosity, particle size, and the recipient site’s biological environment [92]. In the context of personalised grafting, resorbable materials may be advantageous when complete replacement by native bone is desired, such as in minor defects or in situations prioritising natural remodelling.

In contrast, non-resorbable graft materials, such as xenografts and deproteinised bovine bone mineral (DBBM), maintain long-term structural integrity and resist degradation. Their volumetric stability makes them particularly suitable for procedures requiring durable scaffold support, including sinus augmentation, alveolar ridge preservation, and alveolar contouring [106]. Non-resorbable bone allografts, produced through high-temperature sintering, aim to combine the stability of xenografts with the biological familiarity of allogenic matrices, potentially mitigating concerns associated with cross-species materials [22].

Selecting between resorbable and non-resorbable grafts, therefore, requires careful evaluation of the clinical objectives—whether long-term scaffold stability is essential, or whether full integration and eventual replacement by native bone is preferable. Understanding these material-specific characteristics is crucial for the appropriate application of both conventional and CAD-CAM personalised grafts, ensuring optimal regenerative outcomes tailored to each patient’s needs [107].

## 3 | Bone Grafting Mechanisms: Osteogenesis, Osteoconduction, Osteoinduction, and Osteoimmunology

### 3.1 | Osteogenesis: Grafts Forming Bone

Osteogenesis is the process by which living cells in the graft form bone [108]. Autografts are the gold standard of grafts as they include live osteoblast cells that can produce bone matrix directly at the site of implantation. These grafts also elute calcium phosphate and vital proteins during resorption, thereby adding to the regenerative milieu at the defect site. Living cells have maintained their superiority against synthetic substitutes in the repair of critical-size defects, which is attributed to the intrinsic biological effects of the living cells [8]. Both synthetic and allograft grafts are osteo-promotive when combined with growth factors or engineered surfaces, but they seldom replicate the cellular contributions of autogenous tissue.

### 3.2 | Osteoconduction: Guiding the Formation of New Bone

Osteoconduction is a three-dimensional process of bone formation within a porous structure implanted in or next to existing bone [8]. Here, the graft functions as a passive matrix, creating sufficient space for capillaries and osteoprogenitor cells to migrate in and induce new bone formation. Some factors influence the osteoconductivity of the graft material. Special attention is paid to porosity and microarchitecture, with pore sizes ranging from 0.7 to 1.2 mm. This is also recognised as optimal for receiving enough vascularity, which is necessary and vital [109]. Surface properties (e.g., roughness) are also important, as evidence suggests that a submicron surface texture on calcium phosphate-based materials, such as hydroxyapatite or tricalcium phosphate, improves osteoblast differentiation and attachment compared with smoother surfaces. Additionally, the material type affects osteoconductivity. Titanium and titanium dioxide, for instance, are well known for their biocompatibility and structural properties, making them suitable for load-bearing applications [6, 110–116]. On the other hand, TCP, as bone void fillers, finds wide application due to its capability to function as a scaffold for osseous ingrowth, leading to its resorption and replacement by host bone in vivo [107].

### 3.3 | Osteoinduction: Mechanisms of Inducing Osteogenesis

Osteoinduction, on the other hand, is a more dynamic biological interaction in which the undifferentiated mesenchymal cell is induced to differentiate into an osteoblast [117]. Bone morphogenetic proteins (BMPs), along with other growth factors, are key signalling molecules in osteogenic induction pathways. These molecules trigger and regulate cellular processes, leading to osteoblast differentiation and subsequent bone matrix formation. Furthermore, the positive and negative effects of physical stimuli, including ionic, electric, magnetic, or mechanical signals, on the osteoinduction of hMSCs have been described, attributed to the modulation of intracellular calcium concentration, which can activate or inactivate critical osteogenic pathways [118].

Recent studies on autogenous tooth-derived grafts (ATGs) highlight their potential as a patient-specific osteoinductive source. Yamada et al. [119] demonstrated that processed extracted teeth retain dentine matrix proteins and growth factors, such as BMPs, which can induce mesenchymal cell differentiation and support new bone formation, resulting in predictable regeneration and stable implant placement outcomes [119]. Demineralised bone matrices or synthetic matrix carrier systems are also used to deliver osteoinductive proteins, such as BMP-2. By a prolonged, slow release of these factors, these carriers could enhance and intensify their local effects, thereby making the new bone formation process more effective and focused [109]. Osteoinduction relies on signalling molecules that stimulate progenitor cells to differentiate into osteoblasts. Beyond BMP-2, several other factors contribute: BMP-7 promotes stem cell differentiation and matrix production [120–123]; TGF-β family members regulate early proliferation and matrix deposition [124]; PDGF enhances chemotaxis and supports early angiogenesis [125, 126]; and VEGF is essential for neovascularisation, coupling angiogenesis with subsequent bone formation [127, 128]. Demineralised bone matrices and synthetic carrier systems are widely used to deliver such osteoinductive proteins. By providing a controlled, sustained release, these carriers help prolong local signalling activity and thereby strengthen and focus the bone-forming response [129–131]. In addition to endogenous proteins, synthetic osteoinductive peptides offer a bioactive, low-immunogenic alternative. P15, a collagen-mimetic peptide, promotes cell adhesion and osteoblast differentiation [132, 133], while P2 enhances early osteogenic signalling and improves scaffold–cell interactions [73, 75, 77, 134]. These molecules, peptides, and delivery systems create a coordinated environment that supports cell recruitment, vascularisation, and osteogenic differentiation during bone regeneration.

### 3.4 | Graft-Immune System Interactions

**FIGURE 6 |:**
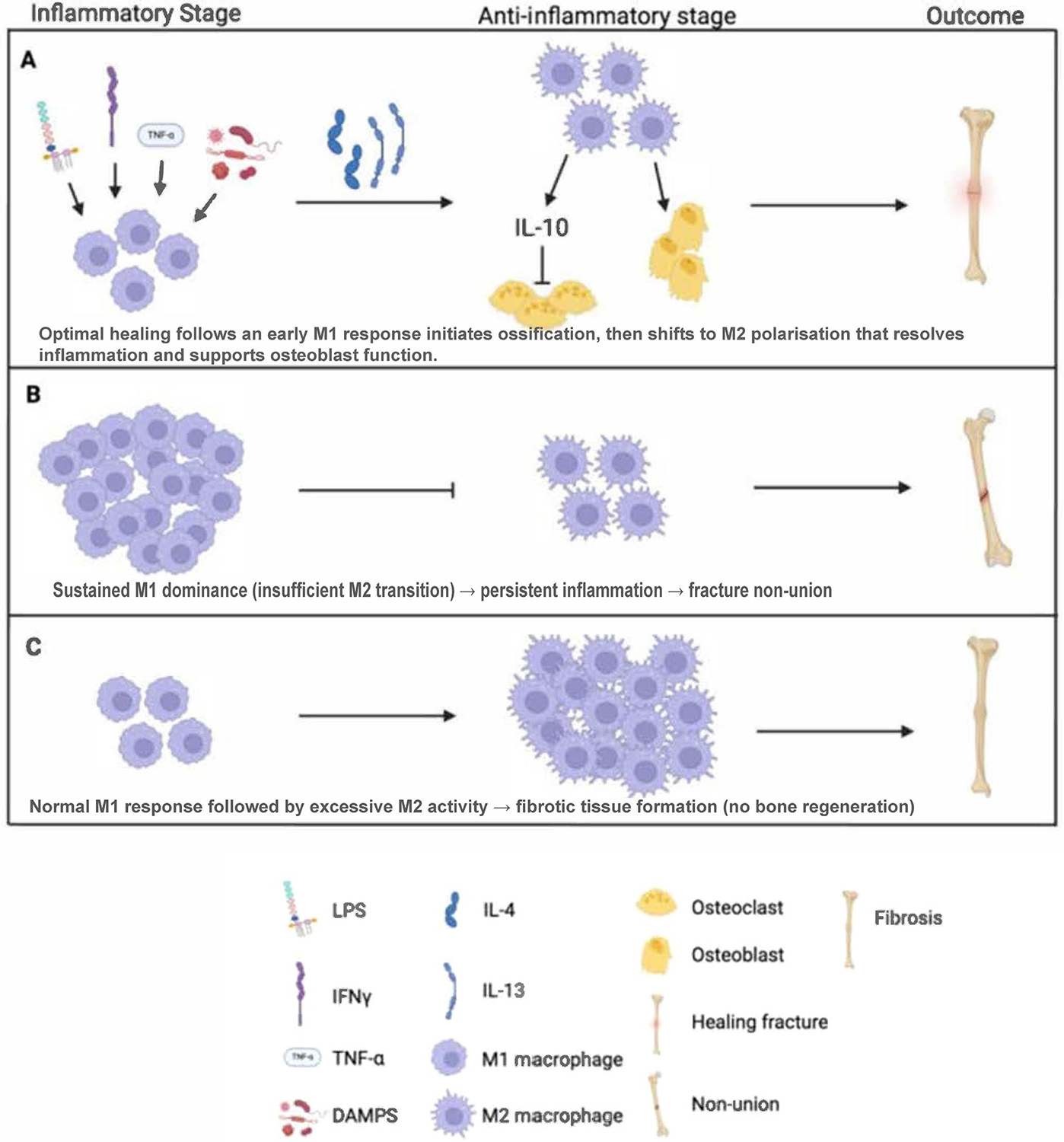
Macrophage polarisation dynamics in bone fracture healing. Illustration depicting the role of M1 and M2 macrophage populations in determining fracture repair outcomes. (A) Optimal healing trajectory characterised by sequential macrophage phenotype transitions: Early M1 macrophage activation (triggered by lipopolysaccharide, damage-associated molecular patterns, interferon-gamma, and tumour necrosis factor-alpha) promotes initial intramembranous and endochondral ossification. Subsequent M2 polarisation, mediated by interleukin-4 and interleukin-13 secretion, generates anti-inflammatory macrophages that produce interleukin-10 to suppress osteoclast activity and neutralise TNF-α, thereby enhancing osteoblast function. (B) Pathological outcome resulting from sustained M1 dominance with insufficient M2 transition: Persistent inflammation leading to fracture non-union. (C) Aberrant healing characterised by excessive M2 activity following normal M1 response: Fibrotic tissue formation rather than bone regeneration [138].

Osteoimmunology examines the intricate relationship between the immune system and bone healing, a relationship that is particularly important to consider during orthopaedic procedures involving allograft or xenograft applications [135]. Osteoblast function can also be negatively affected by inflammatory mediators, particularly cytokines such as TNF-α and IL-6, which can inhibit key signalling pathways or enhance osteoclastogenesis, thereby accelerating bone resorption [136]. Immunological responses to graft materials, especially when foreign proteins persist, can also lead to fibrotic encapsulation or localised tissue resorption, resulting in a loss of osteoconductive potential. Discoveries in bioengineering materials that tailor immune responses by coordinately designing and tuning physical and mechanical cues, such as specialised coatings or the addition of anti-inflammatory agents, have shown promise in minimising graft complications and promoting bone healing [109]. Therefore, addressing the complex relationships in osteoimmunology could provide ways to improve graft integration and secure durable, stable results in different reconstructive practices [137]. This osteogenic performance underscores the importance of living cells within the graft, making autografts the gold standard for bone repair, despite the inconvenience of graft harvesting. Osteoimmunology remains a rapidly growing area of research, with either inhibitory or pro-regenerative effects on bone regeneration, supporting the concept of immune-controlling approaches for bone graft applications [22, 107]. Recent insights by Jane et al. [138] highlight the central role of innate immune cells, particularly neutrophils and macrophages, in orchestrating the inflammatory and reparative phases of fracture healing. Their review emphasises that a timely shift from pro-inflammatory M1 to regenerative M2 macrophage phenotypes is essential for effective bone repair (Figure 6). At the same time, dysregulation of this transition can lead to delayed healing or non-union. These findings underscore the therapeutic potential of strategies that modulate innate immune responses to optimise graft integration and improve clinical outcomes in bone reconstruction procedures [138].

## 4 | Bone Grafts for Different Clinical Indications: Linking Biological Mechanisms to Clinical Strategy

Successful regeneration is never a matter of simply “filling a void”. Each procedure depends on one or more fundamental biological mechanisms. For instance, when a clinician inserts a particulate xenograft into a fresh extraction socket, that material does not create bone; instead, it passively conducts host cells and vessels into the clot, preserving space until woven bone matures. By contrast, a composite block of autogenous chips and slowly resorbing xenograft placed beneath a titanium-reinforced membrane must do more than preserve contour: it must contribute living osteoprogenitor cells, growth factors, and an early mineralised matrix robust enough to resist micromovement long before complete revascularisation occurs. Appreciating whether a site is cell-rich or cell-poor, whether the periosteum is intact or excluded, and which kind of tissues need to be regenerated (i.e., just bone or whole periodontium) determines whether osteoconduction alone is sufficient or whether the additional attributes of osteoinduction and osteogenesis are indispensable. In the following sections, the principles and evidence supporting the use of different bone grafts across various clinical indications are reviewed (Table 1).

### 4.1 | Periodontal Defects: Space Maintenance Over Cell Delivery

Successful periodontal regeneration also requires the formation of new cementum and periodontal ligament—not merely alveolar bone regeneration. Human histological studies indicate that bone grafts used alone provide limited new connective tissue attachment and do not predictably lead to proper periodontal regeneration [139]. This is mainly because the residual periodontal ligament already contains a rich population of mesenchymal cells and an abundant vascular supply capable of rapid repopulation of the defect. Consequently, bone graft materials contribute little to the delivery of regenerative cells or signalling molecules. Biologics such as enamel matrix derivatives (EMD), recombinant PDGF, and platelet-derived preparations represent the biomaterials of choice, enhancing the intrinsic regenerative potential of the periodontal ligament by stimulating cell recruitment, angiogenesis, and new matrix formation [84]. The primary value of bone grafts in periodontal regeneration lies in space maintenance rather than biological stimulation [140].

Slowly resorbing materials, particularly xenografts and allografts, help prevent early soft-tissue collapse and stabilise the defect during healing. In contrast, autogenous bone—formerly widely used—resorbs rapidly, carries donor-site morbidity, and may increase the risk of root resorption, making it less favourable as a space-maintaining scaffold [139] Increasingly, soft-tissue management strategies (e.g., papilla preservation flaps, minimally invasive approaches) are preferred as first-line methods for maintaining space without graft materials [141–145]. When space-maintaining flaps cannot be performed, combining biologics with a slowly resorbing graft remains a predictable and well-supported approach, especially in non-containing defects, providing both structural stability and a biologically favourable environment for new attachment.

The key clinical evidence on the use of various bone grafts for periodontal regeneration in intra-bony and furcation defects is summarised below. It is important to emphasise, however, that improvements in clinical or radiographic parameters do not necessarily equate to true periodontal regeneration, which can only be confirmed histologically.

#### 4.1.1 | Bone Grafts for Periodontal Regeneration of Intra-Bony Defects

A summary of the evidence available on different bone grafts used in periodontal regeneration of intra-bony defects is provided below:

##### 4.1.1.1 | Autografts.

Clinical studies indicate reduced postoperative gingival recession and a higher proportion of sites achieving substantial clinical attachment level (CAL) gain (≥ 6 mm) when used in conjunction with EMD, compared to EMD alone. In a randomised clinical trial including 40 intraosseous defects, Guida et al. (2007) reported significantly greater mean CAL gain (5.2 ± 1.5 mm) and PPD reduction (5.4 ± 1.6 mm) in the ACBP + EMD group compared to EMD alone (4.0 ± 1.3 mm and 4.5 ± 1.4 mm, respectively; *p* < 0.05), along with lower postoperative gingival recession (0.6 ± 0.4 mm vs. 1.2 ± 0.7 mm). The proportion of sites achieving substantial CAL gain (≥ 6 mm) was 47% with ACBP + EMD versus 27% with EMD alone [146]. An additional effect of autografts on CAL has also been shown for other biologics, such as rhFGF. In a case–control study of 122 defects, Matsuda et al. [147] reported a mean CAL gain of 4.5 ± 1.3 mm and 79% defect fill at 6 months, with healing outcomes strongly influenced by defect depth and morphology. However, not all studies have shown consistent results. For instance, Kojima et al. [148] found no significant improvement when rhFGF-2 was combined with autogenous bone compared with rhFGF-2 alone, with a mean CAL gain of approximately 3.8 mm in both groups.

##### 4.1.1.2 | Allografts.

The adjunctive use of demineralised freeze-dried bone allograft (DFDBA) over EMD has been clinically evaluated. In the randomised clinical trial by Gurinsky et al. (33 defects), EMD + DFDBA achieved a mean PPD reduction of 4.8 ± 1.4 mm and CAL gain of 3.7 ± 1.1 mm, compared to 4.1 ± 1.2 mm and 3.2 ± 1.0 mm for EMD alone. Still, these improvements were not significantly different from those achieved with EMD alone. Conversely, radiographic bone fill averaged 65% in the combination group versus 49% in controls (*p* < 0.05) [149].

##### 4.1.1.3 | Xenografts.

The combination of EMD with bovine-derived xenografts (BDX) has shown enhanced clinical outcomes compared to EMD alone in the treatment of intraosseous periodontal defects. In a randomised clinical trial, Velasquez-Plata et al. [150] reported significantly improved defect fill (4.0 ± 0.8 mm vs. 3.1 ± 1.0 mm; *p* < 0.05) and reduced gingival recession (0.3 ± 0.6 mm vs. 0.8 ± 0.8 mm) in the EMD + BDX group compared to EMD alone. In comparison, CAL gain (4.3 ± 1.2 mm vs. 3.9 ± 1.1 mm) and PPD reduction (5.0 ± 1.4 mm vs. 4.7 ± 1.3 mm) were comparable between groups. Complementary findings by Zucchelli et al. [151] in 30 deep intraosseous defects treated with a minimally invasive approach showed that this combination resulted in significantly greater CAL gain (3.1 ± 1.0 mm vs. 2.1 ± 1.2 mm; *p* < 0.05), superior defect resolution (79% vs. 62%), and improved soft tissue stability compared to EMD alone. Histological data and 12-month radiographs confirmed 65%–80% defect fill, suggesting enhanced space maintenance and clot stability when EMD is combined with bovine xenografts [151].

##### 4.1.1.4 | Alloplasts.

Synthetic bone substitutes have been explored as space-maintaining scaffolds in combination with EMD. De Leonardis and Paolantonio (2013) randomised 48 defects to EMD alone or EMD + biphasic calcium phosphate (BCP). After 12 months, both groups showed significant improvements, but the combination group achieved superior PPD reduction (4.2 ± 0.5 mm vs. 3.1 ± 0.8 mm; *p* < 0.01), CAL gain (3.5 ± 0.7 mm vs. 2.4 ± 1.0 mm; *p* < 0.01), and radiographic bone fill (82% vs. 60%) [152].

In comparing different bone grafts for periodontal regeneration surgery, Kao et al. reported that combining EMD with bone grafts generally enhances clinical outcomes, with autografts and allografts showing slightly superior CAL and bone fill compared with xenografts and alloplasts. However, the review highlighted that evidence of histologic true regeneration (new cementum, PDL, and bone) remains limited [153].

Overall, current evidence supports that all bone graft types—autogenous, allogenic, xenogeneic, and alloplastic—can enhance the regenerative potential of EMD in intra-bony defects, primarily by improving defect stability and soft tissue architecture. Material selection should therefore be guided by defect morphology, handling properties, morbidity, and patient preference, rather than by a presumed biologic hierarchy among graft types, although non-autogenous grafts may be preferred, given the preclinical evidence of micro–root resorption reported with autogenous grafts [154].

#### 4.1.2 | Bone Grafts for Periodontal Regeneration of Furcation Defects

The treatment of furcation defects remains a clinical challenge due to complex anatomical morphology and limited access for instrumentation. The use of bone grafts to maintain space—whether combined with resorbable membranes as part of guided tissue regeneration or with enamel matrix derivatives—has demonstrated clinical effectiveness in Class II furcations, particularly in mandibular molars, although complete furcation closure remains uncommon.

A recent Bayesian network meta-analysis of randomised clinical trials (Jepsen et al., 2020), including 20 studies and 787 furcation-involved molars, confirmed the superiority of regenerative surgery over open flap debridement (OFD) in furcation defects [155]. Regenerative approaches yielded a mean horizontal clinical attachment level (HCAL) gain of 1.6 mm, vertical CAL gain of 1.3 mm, and probing pocket depth reduction of 1.3 mm compared with OFD. Furcation closure rates varied widely across studies, ranging from 0% to 60%, while Class II-to-Class I conversion occurred in up to 100% of sites, depending on the technique used. Treatments involving bone grafts—including autografts, allografts, and xenografts—achieved the highest probability (61%) of being the best option for horizontal bone level (HBL) gain, followed by resorbable membranes combined with grafts (51%). Combination therapies, such as GTR + bone grafts ± EMD, achieved furcation improvement (closure or conversion) in 86%–100% of treated sites, compared with 6% in OFD controls.

Overall, these results highlight that bone grafts play a key role as space-maintaining and osteoconductive materials in the regenerative surgery of Class II furcations. When combined with biologics such as EMD or barrier membranes, they can enhance furcation closure and CAL gain.

### 4.2 | Socket Preservation: Maintaining Ridge Contour After Extraction

Although bone remodelling after extraction cannot be avoided (especially on the buccal bone wall), it can be significantly diminished by the proper use of bone grafts through socket preservation procedures. Because the socket is still surrounded by vascular cancellous bone, osteogenesis from the graft itself is unnecessary.

A recent systematic review and meta-analysis by Avila-Ortiz et al. [156] confirmed that ARP techniques using bone graft materials significantly reduce horizontal and vertical ridge resorption compared to spontaneous healing. Quantitative analysis of 22 RCTs (*n* = 607 patients) demonstrated a mean preservation of 1.99 mm in horizontal width (95% CI 1.54–2.44 mm; *p* < 0.00001), 1.72 mm in vertical mid-buccal height (95% CI 0.96–2.48 mm), and 1.16 mm in vertical midlingual height (95% CI 0.81–1.52 mm) relative to ungrafted controls. The best outcomes were observed in sites grafted with xenogenic or allogenic materials covered with absorbable collagen membranes or sponges, which showed up to 3.2 mm greater preservation when the buccal wall thickness exceeded 1 mm [156]. Although no single biomaterial proved superior, allografts, xenografts, and alloplasts were all effective in attenuating bone loss. However, residual remodelling still occurs despite the procedure. These findings support the routine use of biomaterials when alveolar socket preservation is indicated. Given that osteogenesis is not required and that extractions are often performed without flap elevation, autologous grafts are not considered a first-line option. Instead, several non-autologous graft materials can be employed to limit post-extraction bone remodelling.

#### 4.2.1 | Xenografts

Xenografts, particularly deproteinised bovine bone with collagen (e.g., Bio-Oss Collagen), have shown favourable outcomes in minimising post-extraction ridge resorption. In a randomised split-mouth clinical trial, Araújo et al. [157] reported that after 4 months, grafted sites exhibited only 0.9 mm of horizontal and 0.7 mm of vertical reduction, compared with 3.6 and 1.8 mm, respectively, in ungrafted controls (*p* < 0.001). Although xenografts did not completely prevent resorption, especially of the buccal bone wall, they were effective in preserving socket volume and maintaining ridge architecture.

Alloplastic materials, such as synthetic bone substitutes like medical-grade calcium sulphate hemihydrate (MGCSH), have also shown promising results in socket preservation. In a clinical and histologic study, Aimetti et al. [158] evaluated the healing of intact maxillary extraction sockets filled with MGCSH compared to unfilled controls. After 3 months, the grafted sites exhibited significantly less buccal vertical resorption (0.5 ± 0.3 mm vs. 1.2 ± 0.5 mm) and reduced horizontal ridge loss (2.0 ± 0.6 mm vs. 3.2 ± 0.8 mm; *p* < 0.05). Histologically, vital bone formation averaged 58.8% ± 10.1% in grafted sites versus 47.2% ± 8.6% in controls, indicating greater trabecular maturation and a higher lamellar bone fraction in the apical regions.

#### 4.2.2 | Allografts

Allogeneic bone grafts have demonstrated effective dimensional preservation in post-extraction sites. In a randomised clinical trial by Iasella et al., including 24 patients, tetracycline hydrated freeze-dried bone allograft covered with a collagen membrane reduced horizontal ridge reduction of 1.6 mm and vertical resorption of 2.2 mm compared to unassisted healing controls [159].

The systematic review and network meta-analysis by Canullo et al. provides a comprehensive comparison of biomaterials used for alveolar ridge preservation [160]. Based on 88 RCTs (2805 patients; 3073 sockets), all grafting materials significantly reduced ridge resorption compared to unassisted healing. Xenografts (DBBM, porcine bone) and allografts ranked highest for horizontal and vertical ridge preservation, with mean reductions of ≈1.5–2.0 mm less than controls. Histomorphometrically, platelet concentrates yielded the greatest new bone formation (mean 43%), whereas xenografts had the lowest vital bone fraction (28%–35%) but the highest dimensional stability. No significant differences were observed in subsequent implant survival (≥ 97% at 12 months) or marginal bone levels among biomaterials.

Overall, current high-level evidence supports alveolar ridge preservation using xenografts, allografts, or alloplasts as a predictable means to mitigate post-extraction bone loss, with xenografts providing the most consistent dimensional stability. Material selection should consider anatomical conditions, patient preference, and the targeted timing of implant placement rather than any presumed biologic hierarchy among graft types.

### 4.3 | Maxillary Sinus Floor Elevation

Sinus-floor augmentation illustrates how the same principle is adapted to a different anatomy. Elevating the Schneiderian membrane creates a secluded chamber whose lining maintains an ample blood supply with enhanced osteogenic potential from the membrane itself. The role of biomaterials in sinus lifting relies on their space-maintenance and osteoconductive properties. When sufficient time is provided for the space beneath the membrane, bone will form due to the Schneiderian membrane’s osteogenic potential.

In this context, Trimmel et al. [161] conducted a comprehensive Bayesian network meta-analysis that included 34 randomised clinical trials and 842 sinus augmentations with a healing period of 5–8 months, comparing 28 different grafting materials. Their findings indicated that the mean proportion of newly formed bone (NB%) across studies ranged approximately from 10% to 55%. The highest probability of effectiveness in NB% (surface under the cumulative ranking curve, SUCRA = 81%) was found for bovine xenograft combined with bone marrow concentrate (BMC), followed by bovine xenograft + platelet-rich plasma (PRP) (77%), bioactive glass + autogenous bone 1:1 (70%), nanocrystalline hydroxyapatite in silica gel (70%), and bioactive glass ceramic (70%). Autogenous bone alone ranked twelfth (SUCRA = 57%), showing mean new bone formation values around 39%–46%, whereas pure bovine xenografts yielded approximately 20%–38% new bone formation depending on the study. Significant differences were observed between bovine + BMC and both the allograft and the biodegradable copolymer, favouring the composite graft [161].

These results reinforce the view that xenografts, particularly deproteinised bovine bone, remain the most predictable and widely used materials due to their excellent space-maintaining characteristics and slow resorption rate, ensuring long-term graft stability. Nevertheless, the addition of autologous components, such as bone marrow concentrate, PRP, or autogenous bone itself, may further enhance vital bone formation, especially in large sinus lifts, thereby reducing healing times.

### 4.4 | Ridge Augmentation

Vertical or lateral guided bone regeneration (GBR) presents a significant biological challenge because the graft is deliberately isolated from the periosteum and overlying soft tissues using (non)resorbable membranes or titanium meshes [162]. With the external blood supply temporarily restricted, the construct must provide its own living cellular component for reliable bone gain to occur. In this context, composite grafts (mainly autografts combined with xenografts, guarded by a membrane) are the gold standard procedure reported in the scientific literature when the principles of guided bone regeneration are followed. Indeed, the autograft chips initiate osteogenesis and release osteoinductive proteins. In contrast, the xenogeneic matrix counteracts the tendency for rapid resorption of pure autogenous bone, helping to maintain the designed contour throughout healing and reducing morbidity (4).

Calciolari et al. [163] conducted a comprehensive Bayesian network meta-analysis including 36 articles (23 clinical trials, 20 RCTs, and 3 CCTs) with more than 500 treated sites, comparing various membranes, bone substitutes, and bioactive factors used in lateral GBR procedures. Across studies, the mean horizontal ridge gain ranged between 3.5 and 5.5 mm, with xenograft + autogenous bone mixtures showing the most stable dimensional outcomes and the lowest resorption rates (mean resorption ≈18%–22% at 6–12 months). SUCRA analysis ranked DBBM + collagen membrane as the most effective combination for lateral augmentation (SUCRA ≈81%), followed closely by DBBM + autogenous bone + collagen membrane (SUCRA ≈78%). Allografts produced similar horizontal gains (3.8–5.0 mm), but with greater interstudy variability, whereas alloplasts performed adequately when used in composite grafts. Importantly, regardless of graft material, outcomes were strongly influenced by primary closure, graft fixation, and membrane integrity, underscoring the procedural sensitivity of GBR rather than a purely material-dependent effect [163].

Conversely, randomised clinical trials comparing different bone grafts for vertical bone augmentation remain limited. In a split-mouth pilot trial, Fontana et al. observed no clinical or histological differences between autogenous and allogenic bone chips used beneath a titanium-reinforced e-PTFE membrane [164]. Similarly, Merli et al. evaluated the Fence Technique in a double-blind RCT comparing 100% autogenous bone versus 50% DBBM + 50% autogenous bone in 30 patients with severe vertical defects. At 6 months, the vertical bone gain averaged 3.7 mm ± 1.5 for the composite graft group and 2.2 mm ± 1.3 for the autogenous bone group (*p* = 0.038), while the volumetric bone increase was 869 mm^3^ vs. 648 mm^3^, respectively, without significant differences in complication rate or implant survival [165]. The companion histologic analysis of this trial confirmed comparable percentages of newly formed bone (≈30%–35%), but with significantly more residual graft particles in the composite group (*p* = 0.031) [166].

These findings collectively suggest that xenograft–autograft mixtures provide a favourable balance between volume stability and biological performance in GBR. While autografts remain the gold standard for their osteogenic potential and faster turnover, the addition of slowly resorbing xenogeneic particles minimises graft collapse and donor-site morbidity, thereby representing a clinically advantageous and predictable approach in contemporary GBR protocols.

### 4.5 | Peri-Implantitis Reconstructive Surgery

Non-surgical interventions often fail to achieve successful clinical endpoints in moderate-to-severe peri-implantitis [167]. Therefore, surgical treatment is often necessary. In the presence of intra-bony defects, reconstructive surgical approaches have gained attention for their potential to regenerate lost peri-implant tissues. The ultimate goal would be to achieve re-osseointegration, an outcome that has, however, been demonstrated inconsistently in preclinical models [168].

Alibegovic et al. [169] conducted a multicentre randomised clinical trial involving 138 patients (147 implants) with peri-implantitis and intra-bony defects ≥ 3 mm, randomised to either an access flap alone or to reconstructive surgery using collagenised bovine bone (DBBM-C). At 3 years, both groups showed significant improvements in probing pocket depth (PPD) and marginal bone level (MBL), with mean reductions in PPD of approximately 3.7 mm and gains in MBL of approximately 1.0 mm. However, only 16.4% (test) and 13.5% (control) of implants fulfilled the composite success endpoint (implant retained, PPD ≤ 5 mm, no BOP/SOP, and ≤ 1 mm recession). No significant between-group differences were found, but buccal recession was less pronounced in the test group (0.7 ± 0.9 mm vs. 1.1 ± 1.5 mm). In the 1-year report of the same trial, Derks et al. [170] published similar findings, showing that the bone graft did not improve PPD or radiographic outcomes. However, soft-tissue recession was significantly smaller in grafted sites. Long-term evidence remains limited, but the 10-year follow-up by La Monaca et al. [171] provides valuable insight. In 34 implants with peri-implantitis associated with intra-bony defects ≥ 3 mm and treated with a mineralised dehydrated bone allograft (MDBA) and resorbable membrane, mean MBL improved from 4.78 ± 1.84 mm at baseline to 3.10 ± 1.73 mm after surgery, and stabilised at 3.71 ± 1.78 mm at 10 years, with a cumulative success rate of 53%–58% according to composite and primary outcomes.

While bone grafts may confer only subtle clinical advantages in the treatment of peri-implantitis, it is important to note that—contrary to other indications such as periodontal regeneration and guided bone regeneration—the literature consistently shows no added benefit from combining additional biomaterials with bone grafts. For instance, three recent randomised clinical trials have shown no additional clinical benefit from combining biomaterials such as enamel matrix derivative (EMD) or resorbable membranes with bone grafts [172–174]. Regidor et al. observed that the adjunctive use of a collagen membrane did not enhance composite disease resolution (≈40% in both groups) [172]. Monje et al. found that adding a membrane to xenogeneic grafts did not improve PPD reduction or bone fill (mean PPD reduction ≈3.5–4.0 mm in both groups) [173]. At the same time, Regidor et al. showed that adding EMD to reconstructive therapy did not increase re-osseointegration or bone gain, with similar radiographic improvements of 0.8–1.1 mm in both EMD and control groups [174].

When comparing different bone grafts for peri-implantitis treatment, no single biomaterial has been shown to outperform others in terms of clinical resolution or bone gain [175, 176]. Indeed, the choice of graft material plays a secondary role to factors such as prompt diagnosis [177, 178], control of risk factors [179–183], decontamination efficacy [184], defect configuration [184, 185], and maintenance protocols [186] than on the choice of graft material alone.

## 5 | The Ideal Bone Graft: Challenges and Emerging Strategies

**FIGURE 7 |:**
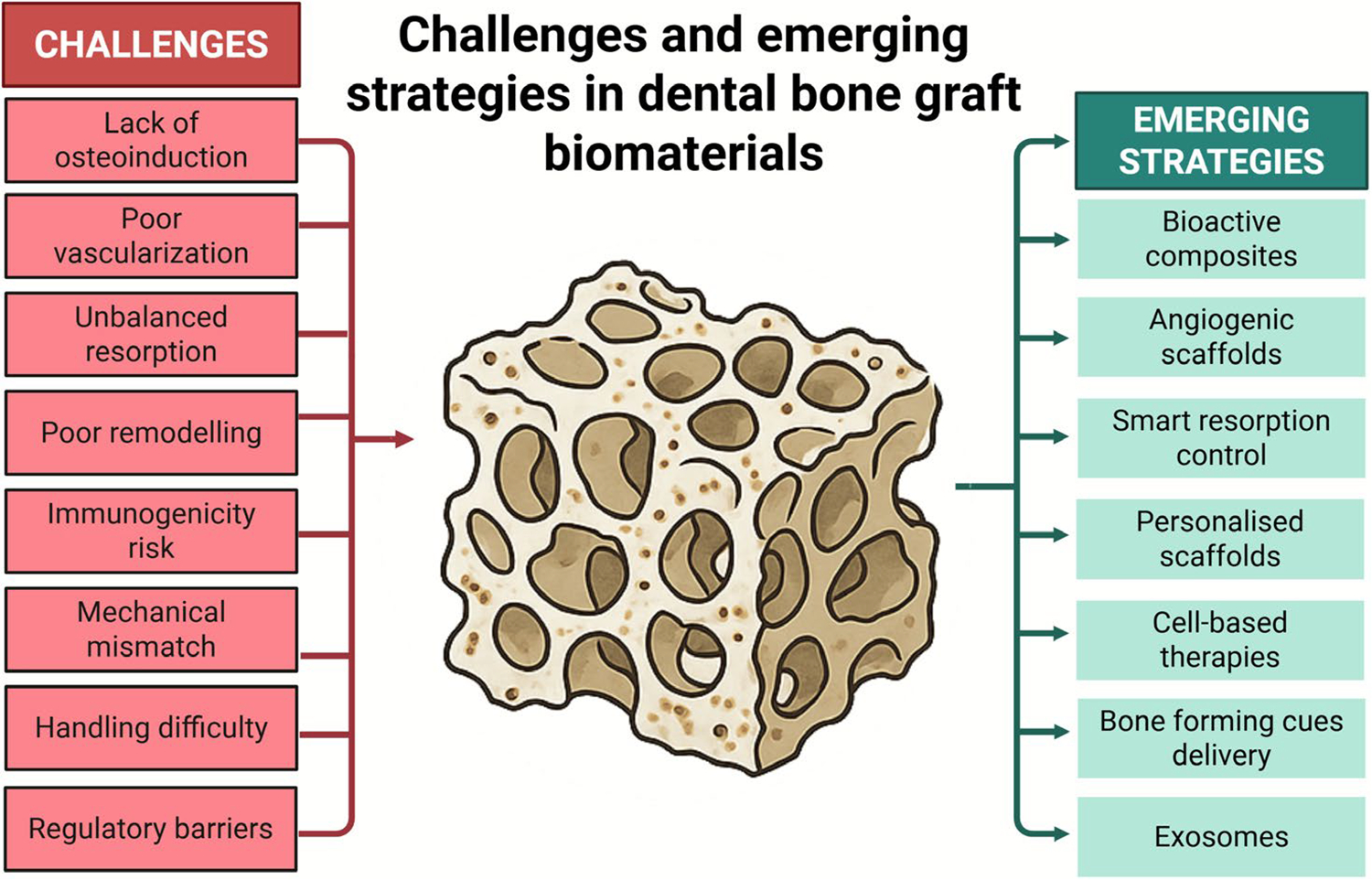
Challenges and emerging strategies in dental bone graft biomaterials. Illustration summarising key limitations of current grafts—such as poor vascularisation, limited osteoinduction, and unbalanced resorption—and emerging approaches including bioactive and angiogenic scaffolds, controlled resorption, and cell-based or exosome-mediated therapies to enhance bone regeneration.

Designing the ideal bone graft remains a significant challenge [187]. Bioceramics, such as hydroxyapatite (HA), replicate the mineral phase of bone but are prone to fracture under functional loads [188]. Polymers can be moulded into virtually any shape, but their mechanical properties are poor [189, 190], and the acidic degradation products can irritate the host tissue [191, 192]. Metals, responding to occlusal load, “over-protect” the site and cause stress shielding that can precipitate marginal resorption in areas of dense bone contribution [193]. Balancing degradation with regeneration further increases complexity: a graft that remains too long may acidify its environment, whereas one that degrades too rapidly exposes the defect [194, 195]. Finally, the ever-present problem of vascularisation [4, 16] arises. With more massive or irregularly shaped stand-ins, the core may not receive enough blood to facilitate healing, particularly where structural support is needed the most [196]. These interrelated challenges continue to drive innovation in graft design. Composite grafts, which combine organic and inorganic components or blend autogenous bone with synthetic materials, aim to overcome the weaknesses of single-phase systems and achieve a more balanced biological response (Figure 7).

Establishing proper biocompatibility remains one of the most challenging tasks in graft fabrication [197, 198]. Even allogeneic tissue, while convenient, may be immunogenic or hold the remote possibility of pathogen transfer. By contrast, fully synthetic constructs typically lack the molecular “whispers” that coax host cells into a smoothly integrated union [199]. Antimicrobial additives, including those made from silver nanoparticles, reduce the risk of post-operative infection; however, they must be used carefully to avoid increasing cytotoxicity or prolonged inflammation [200–202].

Additionally, complex manufacturing realities further complicate the situation [203]. With three-dimensional printing, we can now mill grafts to the exact dimensions of a patient’s defect [204]; however, replicating the nanoscale-to-macroscopic hierarchy of bone has proven to be significantly more challenging than sketching a surface contour on a screen [105]. Multimodal replacements that combine structure and biology are the standard bearers of approval procrastination, prolonged by the wheels of regulatory rigmarole before they are ready for the chairside. However, research has shifted towards advanced scaffolds, such as nanocomposites and “smart” solutions that respond to the wound environment, as well as approaches to promote the growth of new blood vessels within the graft [18–20]. Autogenous bone, long hailed for its osteogenic, osteoinductive, and osteoconductive benefits, remains the gold standard; however, ongoing improvements in the field, particularly with next-generation substitutes, derived from xenogeneic, allogeneic tissue, or manufactured from ceramics, polymers, and their composites, aim to mimic the performance of the patient’s bone, thereby avoiding such clinical limitations [22]. Table 2 summarises the main challenges, including insufficient osteoinductivity, limited vascularisation, unbalanced resorption–formation dynamics, and poor mechanical or biological integration, which lead to variable clinical outcomes and limited translational potential. This emphasis on ongoing improvements should instil a sense of hope and progress in readers.

## 6 | Bone Grafts: Innovations and Future Trends

### 6.1 | Smart and Responsive Biomaterials

Recent advances in composite bone graft materials/systems indicate a growing trend towards biomaterials that interact with the biological environment rather than providing only structural support [205]. Among them are shape-memory polymers (SMPs) combined with bioactive ceramics, which can be used to prepare grafts with appropriate mechanical properties over multiple time periods, responding to the process of bone healing. Meanwhile, bioreactive drug-delivery strategies responsive to local biological conditions have been developed for the controlled release of osteogenic or anti-inflammatory factors, thereby maximising therapeutic efficacy while minimising systemic administration. Today, composites are being engineered with machine learning and artificial intelligence tools for predictive modelling to enable rapid material screening, performance optimisation, and patient-specific design [206].

Such composite scaffolds, with controlled drug release, further enhance therapeutic effects beyond mere structural support [62, 207]. Methods based on systems that trigger the release of bioactive ions or molecules to stimulate osteoblastic activity and new bone formation have also been described, with effects up to 100-fold [208, 209] Some promising fields of study include ‘smart’ biomaterials. These are materials that can feel or respond to biological or mechanical cues at the site of injury. They can respond to local signalling and be modified to deliver growth factors and an anti-inflammatory molecule locally. The possibilities of these materials are so exciting and could pave the way for a whole new era in regenerative medicine [63]. Such a principle has already been employed in hybrid scaffolds with electro-magnetic cues (galvanotaxis) guiding cell migration towards defect sites.

In the era of personalised regenerative medicine, these advances are accompanied by a knowledge of graft gene expression profiles and proteomic (as well as phenotypic) characterisation to rationalise optimal graft selection in the latest evolution, including genomic contributions [210]. So, all that is very reassuring, and it speaks again to the importance of patient-specific planning based on computational modelling. This can be used to predict person-specific healing progress, thereby increasing the predictability of treatment and long-term outcomes [211].

### 6.2 | Combination of Bone Grafts With Biological and Cell-Free Therapeutic Innovations

The world of bio is imitating the unliving. Recombinant growth factors, such as rhBMP-2 and rhPDGF, have been combined with calcium phosphate carriers to enhance osteogenesis without donor-site morbidity [62, 63, 67, 68]. Supraphysiological dosing, however, has raised concerns about potential adverse events [60, 61], driving initiatives to develop safer alternatives, such as proline-rich intrinsically disordered peptides (IDPs), P2 and P6 [78, 134, 212]. These peptides mimic the receptors of short-lived extracellular matrix proteins, thereby promoting long-term osteoblast proliferation and mineralisation [73, 74, 76]. It is worth noting that although the progress is promising, it carries potential risks and limitations that should be carefully weighed in clinical practice.

Among the technological developments over the years, biologically active membrane and extracellular vesicle technologies are also critical advancements for the development of composite grafts, alongside molecular engineering [213–215]. The HAM is a layer of membrane derived from the human placenta that is naturally enriched in ECM with a high content of collagen, as well as adequate amounts of growth factors (VEGF, TGF-β1) and anti-inflammatory properties [216, 217]. Fenelon et al. [218] demonstrated that an HAM combined with a calcium-phosphate scaffold, applied incorporating rhBMP-2, facilitated bone regeneration at the femoral critical-sized defect in one operation, with rates similar to those of the two-stage Masquelet-induced membrane technique [218]. HAM is a bioactive, clinically effective alternative that could minimise patient morbidity, operative time, and cost without compromising regenerative potential.

Concurrently, there is intensive focus on cell-free therapies, including mesenchymal stem cell–derived exosomes (MSC-Exo) [215]. As described by Torrecillas-Baena et al. [219], MSC-Exo governs all the key stages of bone healing (i.e., inflammation, angiogenesis, proliferation, and differentiation) through nanometre-scale paracrine signalling [219]. These vesicles carry osteogenic miRNAs, proteins, and lipids (lipid rafts), which stimulate osteoblasts and neo-vascularisation within graft sites. Preclinical studies demonstrated that MSC-Exo, when administered intravenously or combined with biomaterials, promoted osteoregeneration in osteoporosis, osteonecrosis, and traumatic defects, thereby addressing immunogenic and ethical concerns associated with living-cell treatments [220, 221].

### 6.3 | Advances in Angiogenesis and Vascular Engineering

The vascularisation process is critical for predictable bone regeneration, and the survival and function of osteogenic cells rely on an adequate supply of oxygen, nutrients, and signalling molecules [222]. In large defects and vertically augmented sites, long diffusion distances prevent recovery from the defects, and insufficient angiogenic ingrowth remains a critical limitation. The existing methods for improving vascularisation include pro-angiogenic growth factors (e.g., VEGF, PDGF), which can be inserted into demineralised matrices or synthetic carriers for controlled release [223–225]. New concepts in scaffold design have further demonstrated that optimising pore architecture, channel orientation, and internal lattice geometry can favour vessel sprouting and perfusion efficiency [226, 227]. Different routes are also used to initiate early vascular induction, including endothelial or progenitor cell communities and emerging bioprinting technologies that can form spatially organised vascular pathways at the tissue-engineering scale [226]. Mechanical loads can affect some factors, including nephronectin (NPNT), VEGF, HIF-1, epidermal growth factor-like domain (EGFL), and Notch ligands. Such factors can regulate the differentiation and proliferation of ECs, encourage bone vascularisation, and improve angiogenic and osteogenic coupling in the local bone microenvironment [228, 229]. From a mechanobiological perspective, manipulating endothelial behaviour to adapt to personalised mechanical signals increases angiogenic–osteogenic coupling, offering novel possibilities for bone grafting [230].

### 6.4 | Synthesis and Outlook

**FIGURE 8 |:**
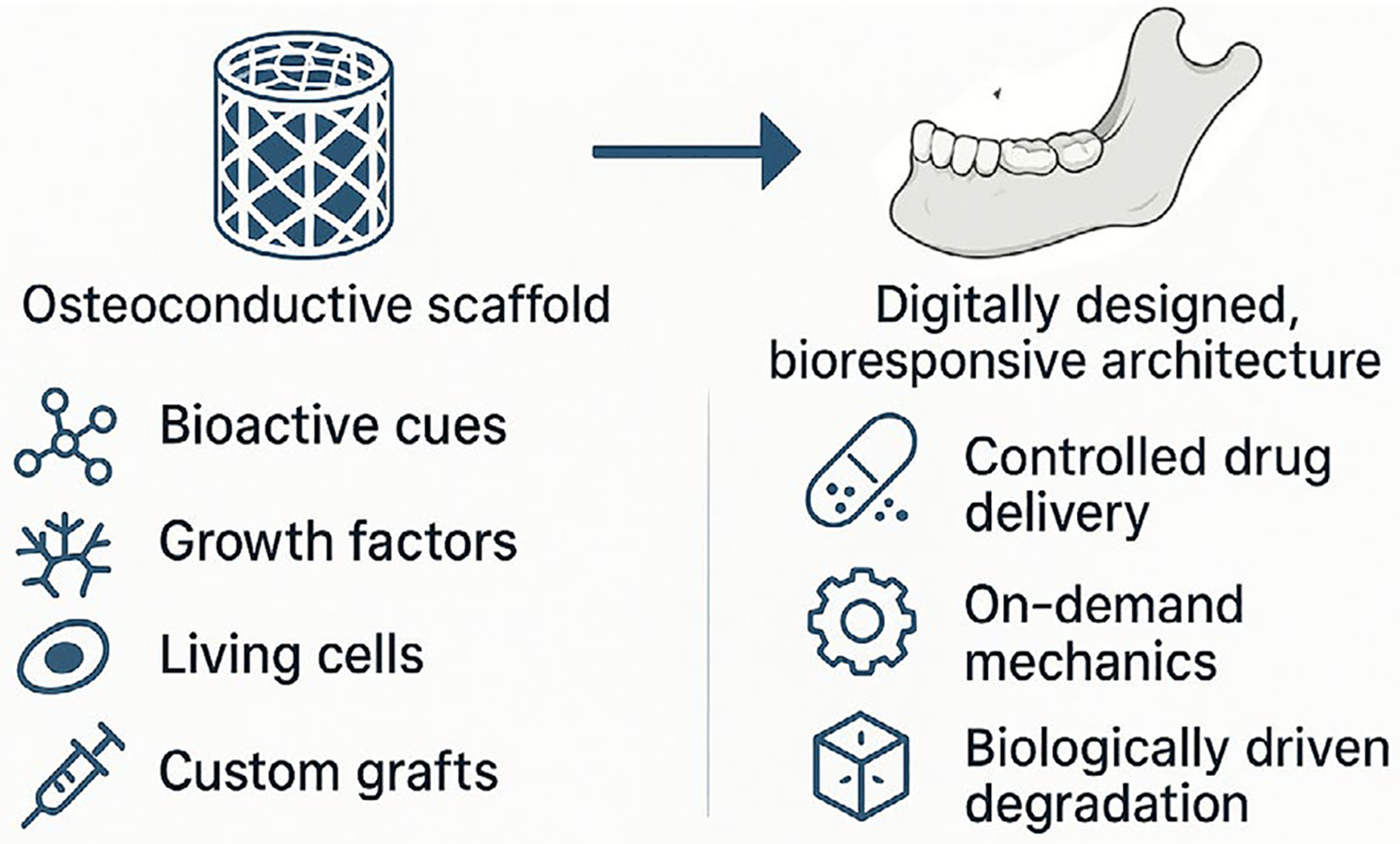
Advances in scaffold design for dental bone regeneration. Conceptual illustration showing the transition from conventional osteoconductive scaffolds incorporating bioactive cues, growth factors, and living cells towards digitally designed, bioresponsive architectures enabling controlled drug delivery, adaptive mechanical behaviour, and biologically regulated degradation.

Together, these complementary advancements—from innovative, response-adaptable scaffolds and molecularly tailored peptides to biologically active membranes and exosome-based therapeutics—herald a renaissance in bioresponsive graft systems with unparalleled precision targeting. The latter systems are aimed not only at substituting for bone volume but also at modulating the biological environment (i.e., degradation/vascularisation and osteogenesis) in a controlled manner. As manufacturing approaches and regulations advance, these new methodologies will alter the future clinical landscape of bone regeneration (Figure 8).

## 7 | Regional, Regulatory and Ethical Considerations

A comprehensive analysis of bone graft materials must also account for the marked regional variations in their clinical use, which are strongly influenced by regulatory considerations, tissue banking practices, and clinician preferences. In North America, allografts are the most common graft option, accounting for the majority of the bone substitute market; however, synthetic alloplasts comprise less than 5% of the volume of placed grafts [19, 231]. This is indicative of a mature, heavily regulated infrastructure for tissue banking, close FDA surveillance, and extensive clinical experience with allogeneic material from human donors. In Europe, perhaps reflecting the more regulated classification of tissue handling prescribed by the European Medicines Agency (EMA) and a legacy of wariness surrounding the use of human-derived grafts following initial concerns about disease transmission, there is overall wider acceptance of xenografts and artificial substitutes. Furthermore, the European market is shifting towards the generation and utilisation of CE-marked synthetic and composite materials, with a regulatory framework that fosters innovation for biomedical applications based on bioceramic systems as well as polymer-based ones. These differences in regulation and culture reinforce how regional governance, market forces, and historical norms shape graft material preferences.

The regulatory and ethical landscape surrounding composite bone grafts represents one of the most complex aspects of modern regenerative medicine, requiring careful navigation of multiple regulatory frameworks, cultural sensitivities, and moral considerations [232]. These materials occupy a unique position at the intersection of medical device regulation, tissue transplantation oversight, and emerging biotechnology governance, creating challenges that extend far beyond simple safety and efficacy evaluations [233]. The complexity is further amplified by the diverse composition of composite grafts, which may include biological components from human, animal, or synthetic sources, each subject to different regulatory pathways and ethical scrutiny [234]. Consequently, harmonising international standards and improving cross-regional regulatory coherence will be essential to facilitate the safe and equitable clinical translation of composite and biomimetic graft technologies worldwide.

### 7.1 | United States FDA Regulatory Pathways

The United States Food and Drug Administration employs a risk-based classification system that categorises bone graft materials according to their complexity and potential for patient harm [235]. The majority of bone graft devices, including many composite formulations, are approved through the 510(k) premarket notification pathway, which requires demonstration of substantial equivalence to legally marketed predicate devices [236]. This pathway, utilised for approximately 94.1% of medical devices in related categories, allows manufacturers to bring products to market without extensive clinical trials, provided they can demonstrate similarity to existing approved materials. A conservative evaluation of this policy shows a 32.9% improvement in the recall rate and a 40.5% reduction in the FDA’s workload. Composite bone grafts containing growth factors or gene therapy components face significantly more stringent regulatory requirements, typically requiring premarket approval (PMA) through clinical trials [233], as classified by the FDA. For example, BMP-2 enhanced composites reflect their increased complexity and potential for systemic effects beyond the local implantation site [236].

### 7.2 | European Union Medical Device Regulation

The European Union’s Medical Device Regulation (MDR), implemented in 2021, has established more stringent requirements for bone graft materials compared to previous regulatory frameworks [233]. Most composite bone grafts are classified as Class III medical devices under the MDR, requiring conformity assessment by notified bodies and comprehensive clinical evidence [237]. The regulation places particular emphasis on post-market surveillance and vigilance reporting, reflecting growing recognition of the need for long-term safety monitoring of implantable materials [233]. The MDR’s approach to classification considers not only the inherent risk of the material but also its intended duration of contact with the body and potential for systemic absorption. Composite grafts incorporating biological components are subject to additional scrutiny under regulations governing materials of biological origin, which require detailed documentation of sourcing, processing, and viral inactivation procedures [238].

### 7.3 | Emerging Technologies and Regulatory Adaptation

The rapid evolution of composite bone graft technologies poses ongoing challenges for regulatory frameworks designed for simpler medical devices [239]. Three-dimensional bioprinting, gene-activated bone substitutes, and smart materials that respond to biological signals require new regulatory approaches that can accommodate their complexity while ensuring patient safety [240]. Current regulatory pathways, developed for traditional medical devices, may not adequately address the unique risks and benefits of these emerging technologies [240]. Artificial intelligence and machine learning applications in composite graft design introduce additional regulatory complexity, as these technologies may continue to evolve after initial approval. The FDA’s Software as a Medical Device (SaMD) guidance provides some framework for addressing these technologies, but their application to composite bone grafts remains largely unexplored.

## 8 | Conclusions

The development of bone grafting materials shows a definite trend towards biomimetic, patient-specific, and biologically interactive systems. Autografts remain the gold standard due to their superior osteogenic, osteoinductive, and osteoconductive properties; however, they entail donor-site morbidity and a limited supply, prompting the ongoing pursuit of effective alternatives. Modern alternatives, whether natural, synthetic, or composite, now generally produce similar results in many clinical situations when defect morphology, vascularity, and surgical stability are appropriately addressed.

Current composites, composed of an osteoconductive scaffold in combination with bioactive signals, growth factors, or even living cells, are advancing beyond space-maintaining tissue towards functional tissue regeneration. Their clinical efficacy results not only from the material’s chemistry but also from tuneable structure (porosity, degradation rate, surface energy), which dictates cell response and integration kinetics. Initial clinical experience already indicates that a printed, injectable, and personalised graft may match the performance of autografts while avoiding donor-site morbidity.

Yet a promising scientific idea alone is not enough for successful translation from the laboratory to the clinic. A clear regulatory pathway and a uniform manufacturing process are crucial for ensuring the safety and efficacy of bone grafting materials. Long-term safety studies and cost-effectiveness remain significant issues for the entire population. In an area that is becoming increasingly crowded, any new material must add a workflow dimension, heal faster, have fewer complications, or require less re-entering surgery to justify its clinical and economic value.

The future of bone regeneration lies in precision biomimetics: digitally designed, bioresponsive architectures tailored to a patient’s anatomy and regenerative profile. These “smart” grafts will combine elements of controlled drug delivery, on-demand mechanical properties, and biologically driven degradation into a unified therapeutic paradigm. Realising this vision will require collaborative advances in materials science, clinical studies, regulation, and industry. When and if these aspects coalesce, comparative biomimicry will no longer be aspirational; composite grafts will exceed autografts, setting a new standard for predictable patient-driven bone regeneration.

## Figures and Tables

**TABLE 1 |:** Overview of bone graft materials in clinical settings.

Indication	Biological challenge	Mechanism required	Recommended graft concept	Autogenous bone
Periodontal regeneration	Collapsing soft tissue, root surface exposed	Space maintenance + cementogenesis	EMD combined with xenograft or allograft for space maintenance	Occasionally in deep defects
Socket preservation	Rapid bundle-bone loss	Osteoconduction, space maintenance	Particulate xenograft or β-TCP; membrane as needed	Not required
Sinus lift	Pneumatised sinus, limited vertical height	Osteoconduction (standard lifts); + osteogenesis if early loading	Xenograft/alloplast alone for staged lifts; ≤ 30% autograft chips when < 4 months healing desired	Only in very large/early-load cases
Ridge augmentation (lateral/vertical)	Cell-poor cortical surfaces, periosteum excluded	Osteogenesis + osteoconduction	30%−70% autograft chips or block mixed with slowly resorbing scaffold, protected by Ti-reinforced PTFE or mesh	Essential
Peri-implantitis reconstructive surgery	Irregular peri-implant defect walls with residual biofilm/granulation tissue after debridement and implant surface detoxification	Osteoconduction	Xenograft or alloplast, when post-surgical recession is an issue, without membranes or biologics (limited added value)	Rarely

**TABLE 2 |:** Key challenges associated with current bone graft materials used in dental and maxillofacial applications.

Challenge	Type of bone graft example	Clinical impact
Lack of osteoinduction	Allografts, ceramics	Slower healing
Poor vascularization	Dense HA blocks	Graft necrosis
Unbalanced resorption	Fast (β-TCP) or slow (xenograft)	Volume loss/persistence
Poor remodelling	Deproteinised bone	Inert remnants
Immunogenicity risk	Allograft, xenograft	Host reaction
Mechanical mismatch	Ceramics, polymers	Suboptimal stability
Handling difficulty	Gels	Surgical inefficiency
Variable outcomes	Calcium phosphates	Unpredictable success
Regulatory barriers	Cell or BMP grafts	Limited translation

## Data Availability

The data that support the findings of this study are available on request from the corresponding author. The data are not publicly available due to privacy or ethical restrictions.
